# Supplementary figures and images for: From roads to biobanks: Roadkill animals as a valuable source of genetic data
Source: PLoS One. 2023 Dec 7;18(12):e0290836. doi: 10.1371/journal.pone.0290836 (PMC10703236; doi:10.1371/journal.pone.0290836)

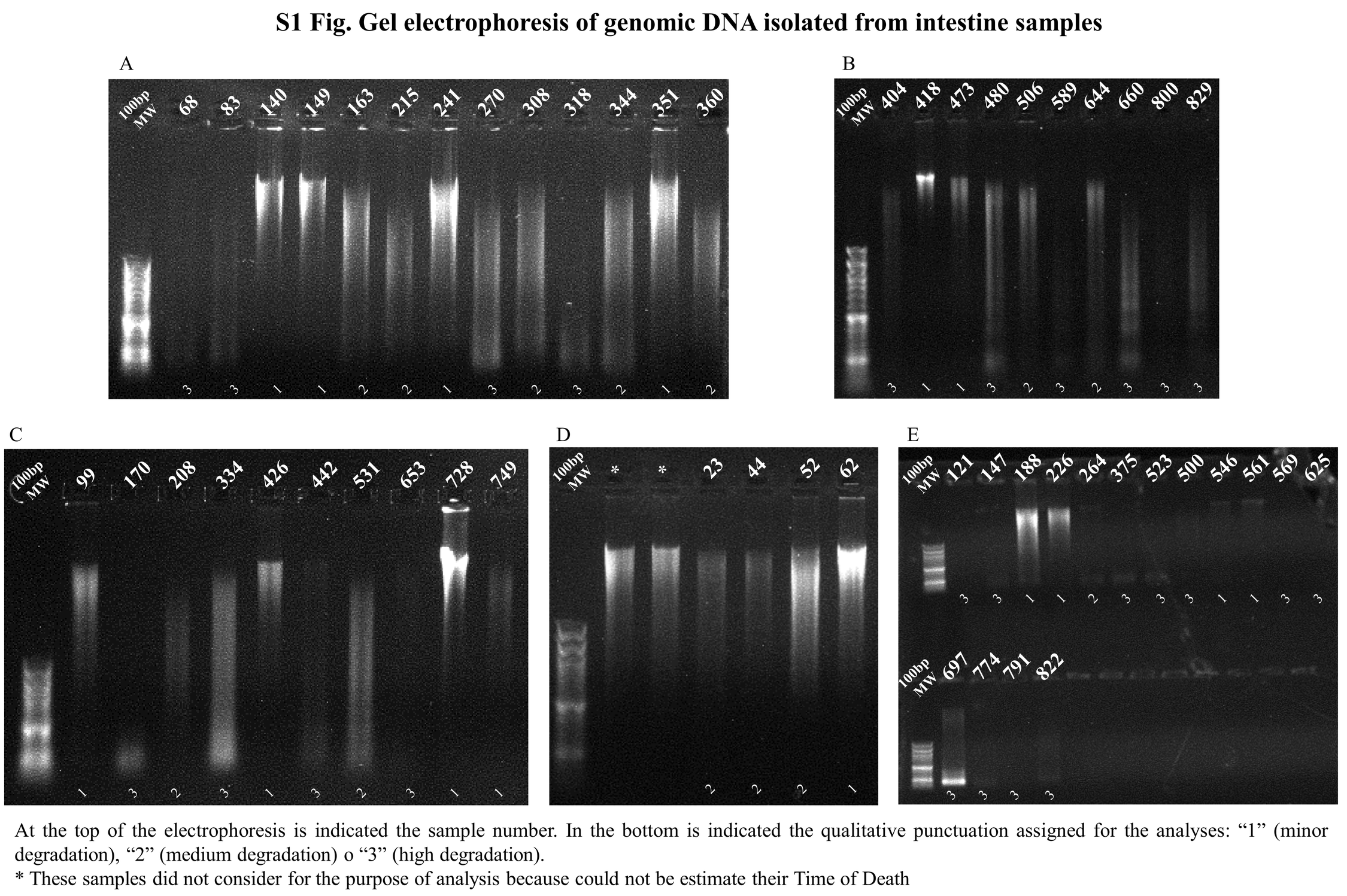

Supplement: S1 Fig — The sample ID is shown at the top. The bottom labels indicate our qualitative classification: “1” (minor degradation), “2” (medium degradation) o “3” (high degradation). (TIF) [file pone.0290836.s001.tif]
