# Supplementary material for: From roads to biobanks: Roadkill animals as a valuable source of genetic data
Source: PLoS One. 2023 Dec 7;18(12):e0290836. doi: 10.1371/journal.pone.0290836 (PMC10703236; doi:10.1371/journal.pone.0290836)
Supplement: S1 Raw images — (PDF) [file pone.0290836.s002.pdf]

S1 raw images for Figure 6

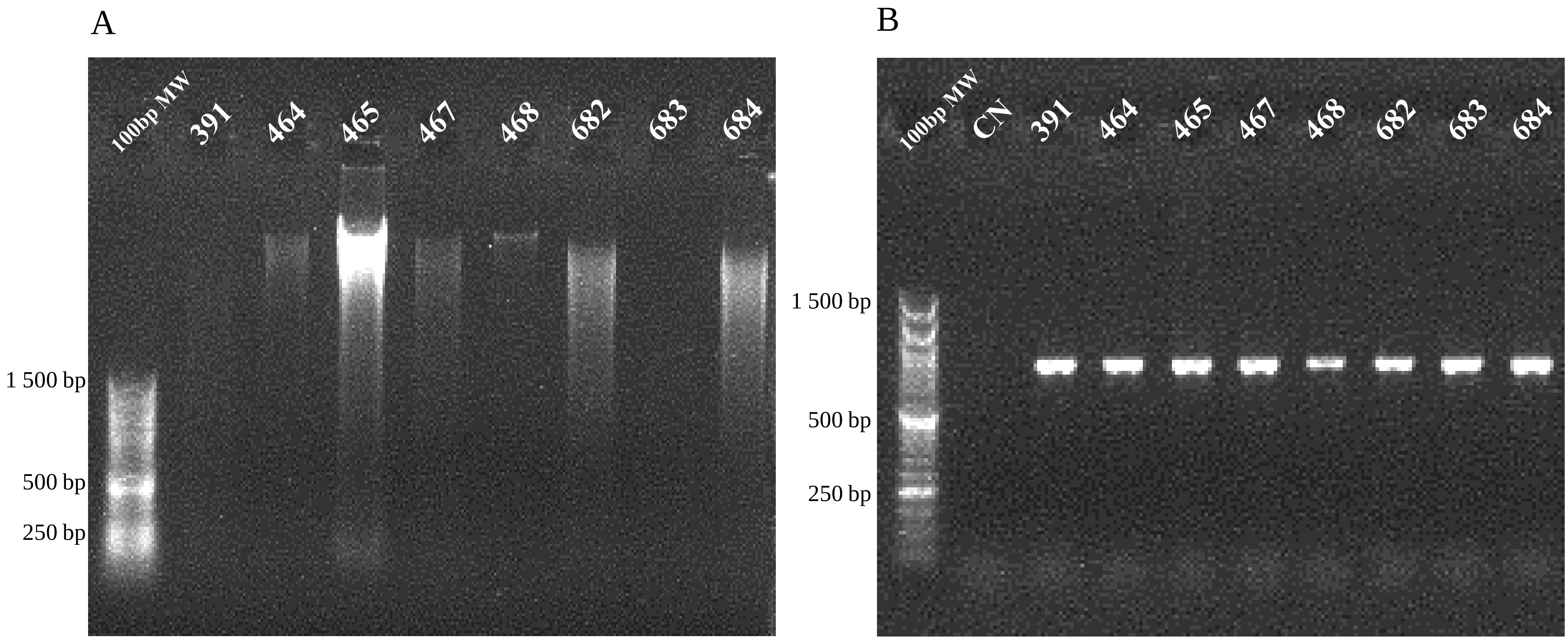

Figure 6. Amplification of vertebrate molecular target. DNA electrophoresis from the indicated samples of vertebrates, containing isolated genomic DNA (A), or the amplified *COI* (B). Molecular weight (MW); Negative control (CN). The number of identification (ID) of each sample is shown in the top line.

S1 raw images for Figure 7

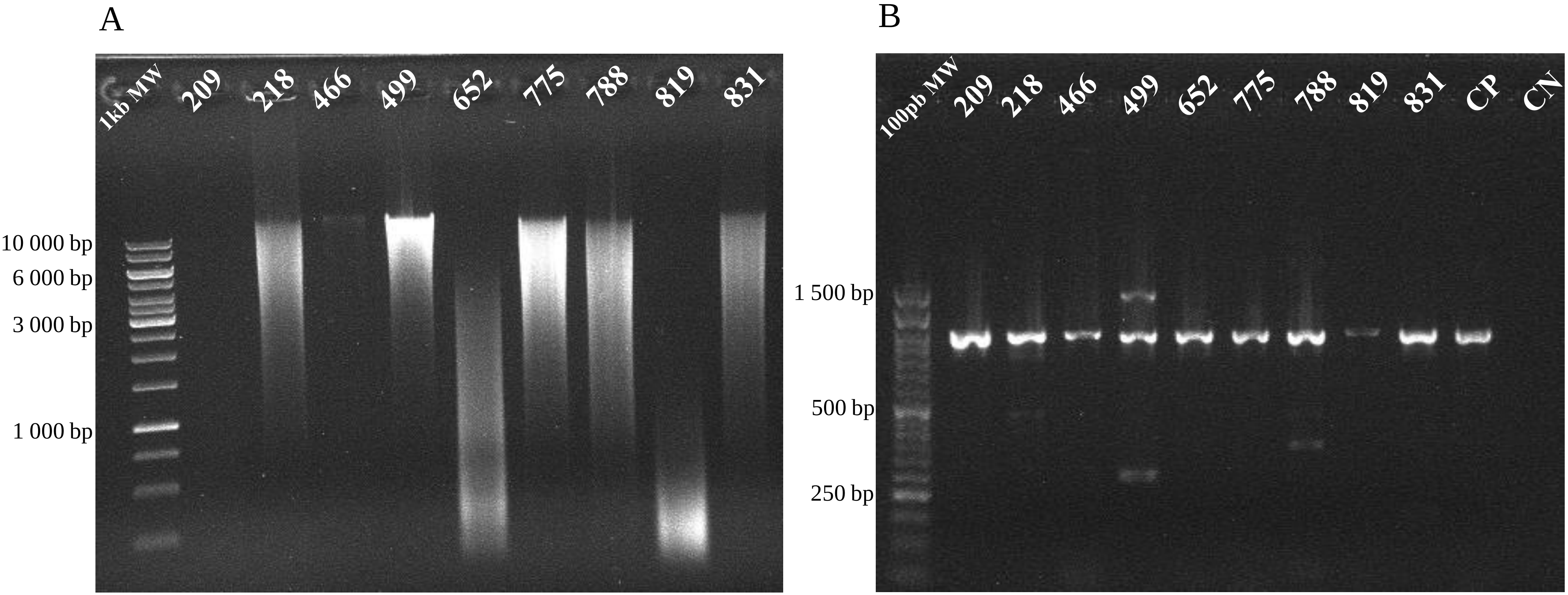

Figure 7. Amplification of molecular target from *Leishmania* spp. DNA electrophoresis from the indicated samples of vertebrate's liver, containing isolated genomic DNA (A), or the amplified *18S SSU rRNA* of *Leishmania* spp. (B).

S1 raw image for S1 Fig

A

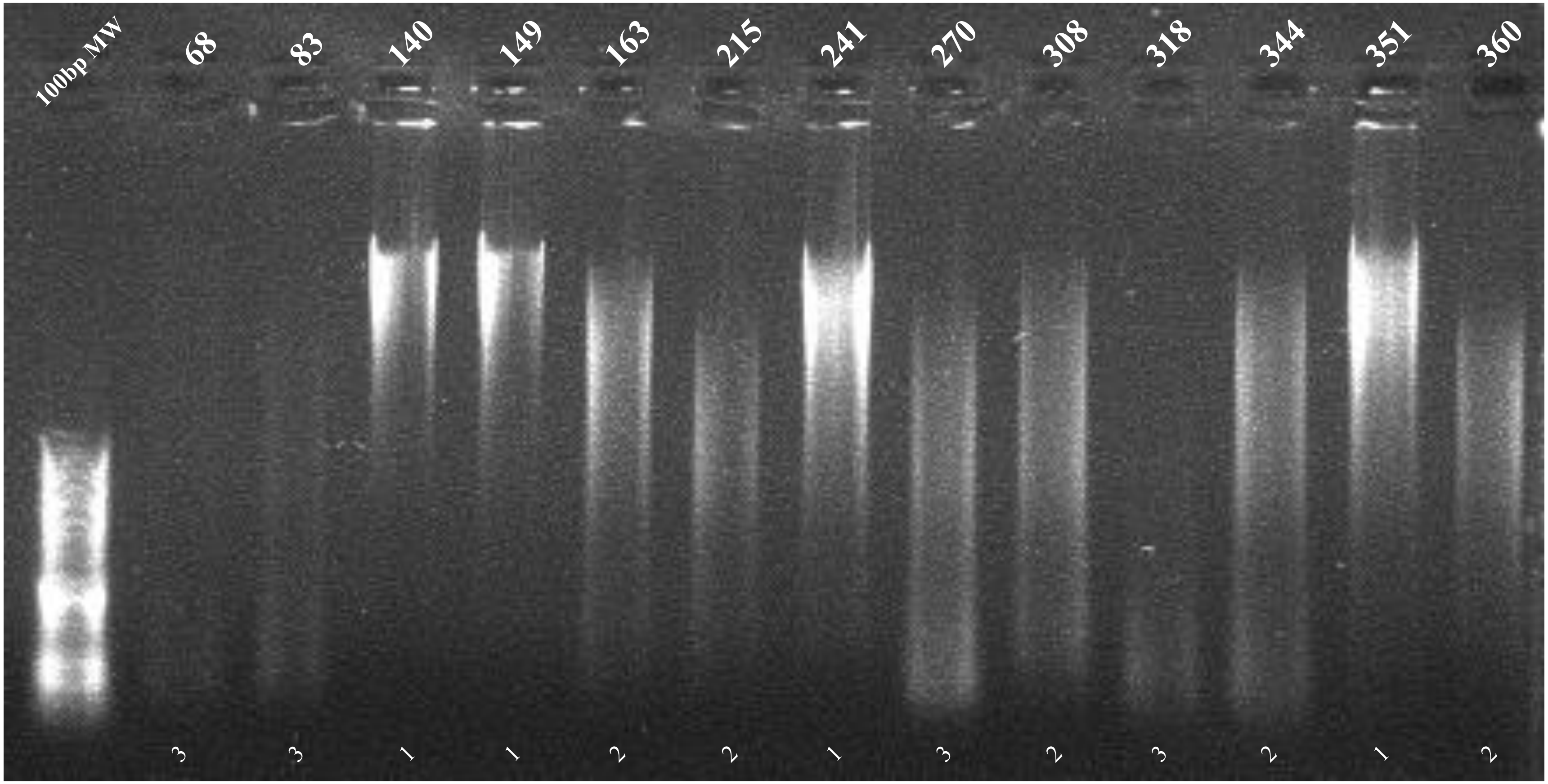

S1 Fig. Gel electrophoresis of genomic DNA isolated from intestine samples. At the top of the electrophoresis is indicated the sample number. In the bottom is indicated the qualitative punctuation assigned for the analyses: “1” (minor degradation), “2” (medium degradation) o “3” (high degradation).

S1 raw image for S1 Fig

B

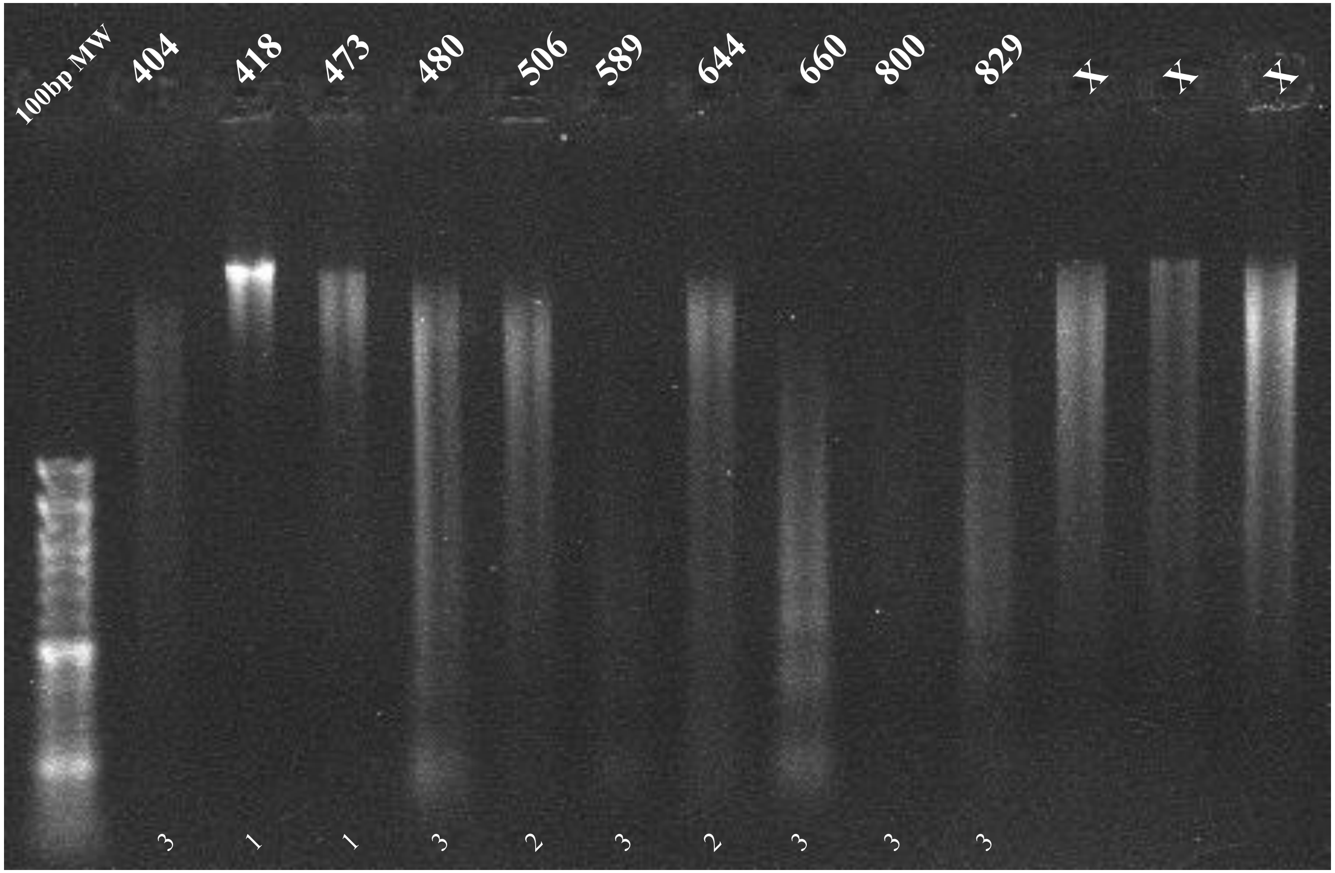

S1 Fig. Gel electrophoresis of genomic DNA isolated from intestine samples. At the top of the electrophoresis is indicated the sample number. In the bottom is indicated the qualitative punctuation assigned for the analyses: "1" (minor degradation), "2" (medium degradation) o "3" (high degradation).

Lanes not included in the final figure are marked with an "X".

S1 raw image for S1 Fig

C

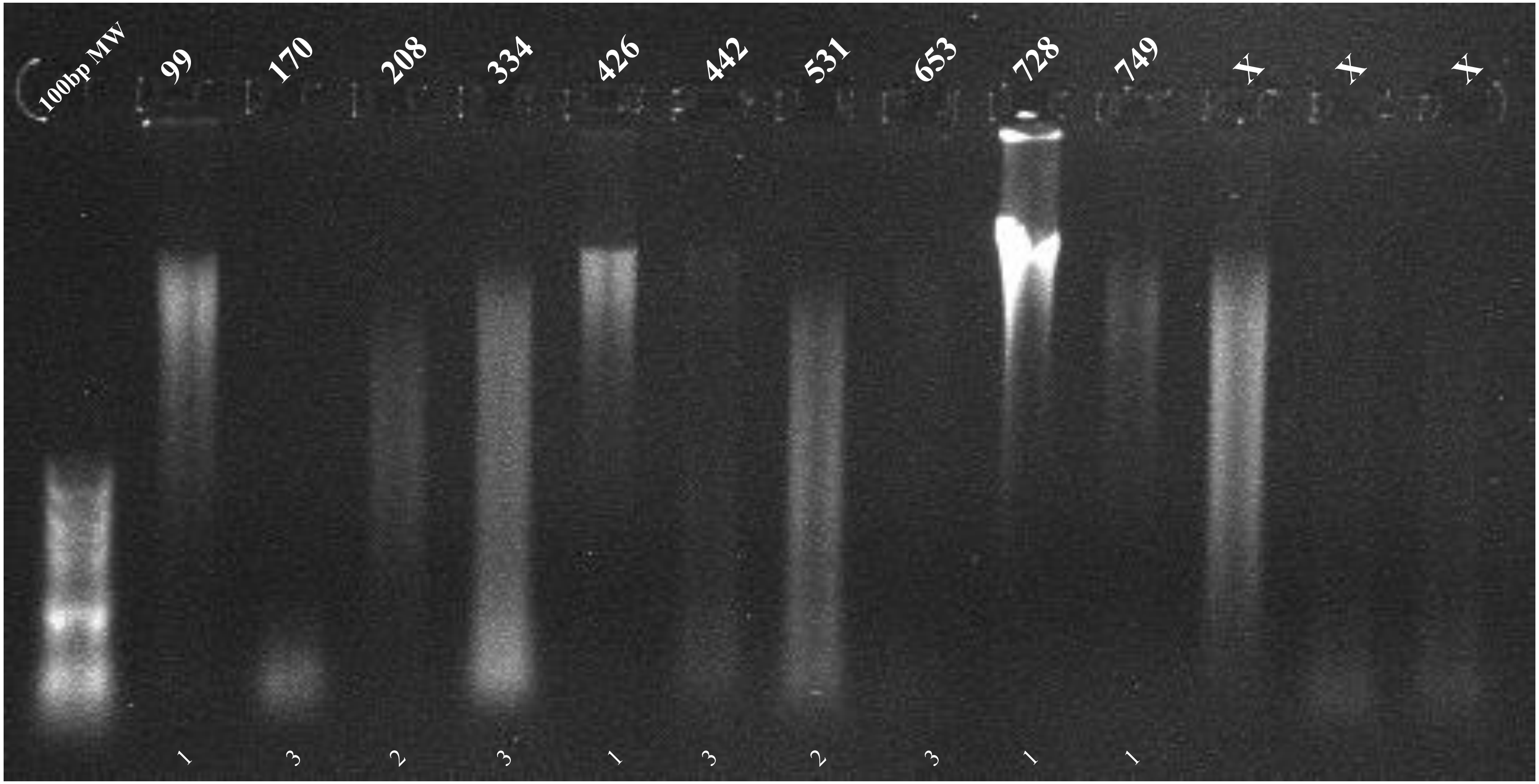

S1 Fig. Gel electrophoresis of genomic DNA isolated from intestine samples. At the top of the electrophoresis is indicated the sample number. In the bottom is indicated the qualitative punctuation assigned for the analyses: “1” (minor degradation), “2” (medium degradation) o “3” (high degradation).

Lanes not included in the final figure are marked with an “X”.

S1 raw image for S1 Fig

D

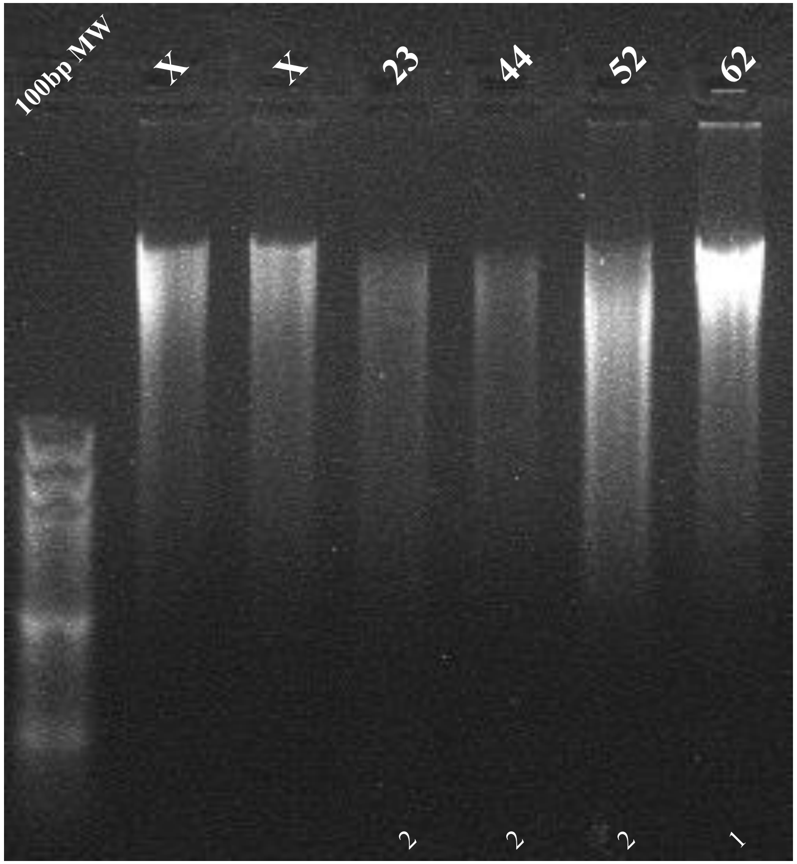

S1 Fig. Gel electrophoresis of genomic DNA isolated from intestine samples. At the top of the electrophoresis is indicated the sample number. In the bottom is indicated the qualitative punctuation assigned for the analyses: “1” (minor degradation), “2” (medium degradation) o “3” (high degradation).

Lanes not included in the final figure are marked with an “X”.

S1 raw image for S1 Fig

E

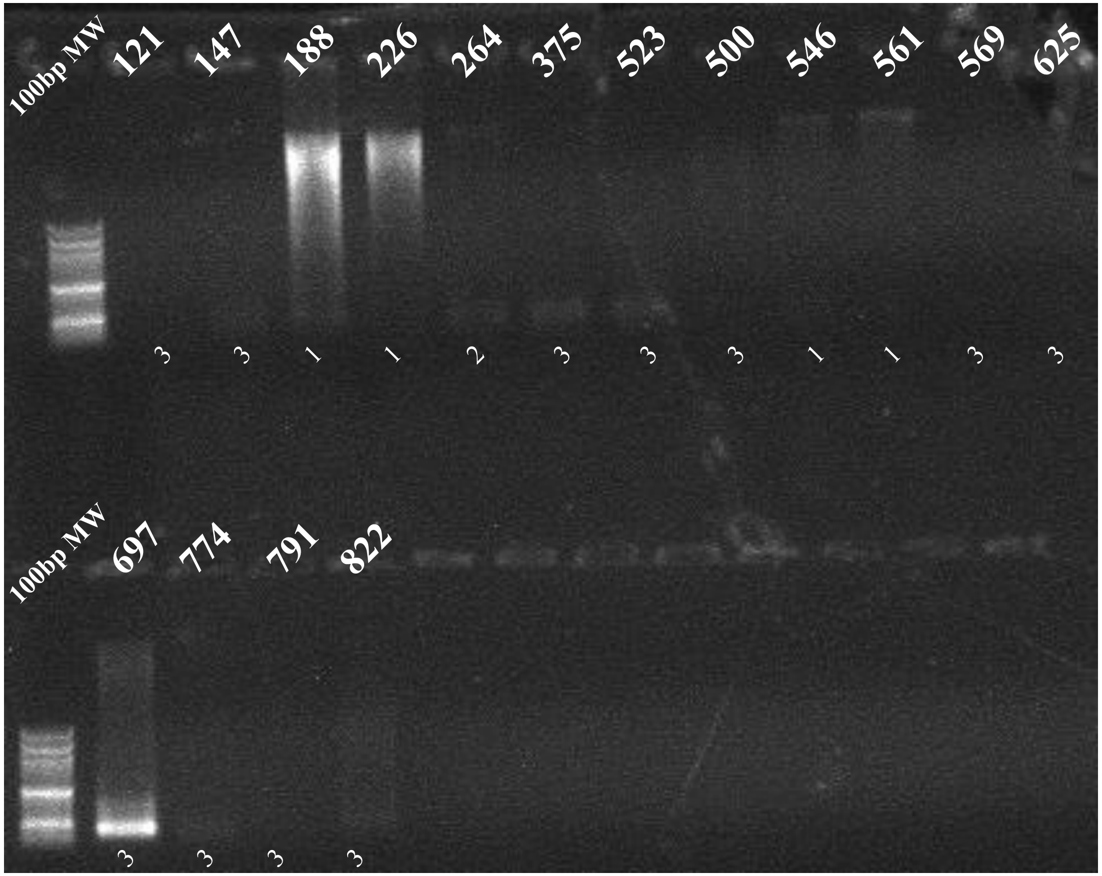

S1 Fig. Gel electrophoresis of genomic DNA isolated from intestine samples. At the top of the electrophoresis is indicated the sample number. In the bottom is indicated the qualitative punctuation assigned for the analyses: “1” (minor degradation), “2” (medium degradation) o “3” (high degradation).
