# Supplementary material for: From roads to biobanks: Roadkill animals as a valuable source of genetic data
Source: PLoS One. 2023 Dec 7;18(12):e0290836. doi: 10.1371/journal.pone.0290836 (PMC10703236; doi:10.1371/journal.pone.0290836)
Supplement: S1 Table — (DOCX) [file pone.0290836.s003.docx]

**S1 Table. Alternative regression models testing how DNA quantity and purity varied by class and time since death.**

| Predictor | Marginal means | SE | 95% CI |
| --- | --- | --- | --- |
| Quantity of DNA (log10) (N=586, R^2^ 0.19) | | | |
| *Linear model with mean DNA per specimen* | | | |
| Amphibia | 1.000 | 0.0518 | 0.903 – 1.110 |
| Reptilia | 1.550 | 0.0388 | 1.478 – 1.630 |
| Aves | 1.590 | 0.0470 | 1.500 – 1.680 |
| Mammalia | 1.710 | 0.0546 | 1.599 – 1.810 |
| Amphibia *TimeDeath* | -0.001 | 0.0027 | -0.006 – 0.004 |
| Reptilia *TimeDeath* | 0.001 | 0.0014 | -0.001 – 0.004 |
| Aves *TimeDeath* | -0.007 | 0.0022 | -0.012 – -0.003 |
| Mammalia *TimeDeath* | -0.004 | 0.0022 | -0.008 – 0.001 |
| *Linear mixed effect models* | | | |
| Amphibia | 1.080 | 0.0979 | 0.830 – 1.340 |
| Reptilia | 1.660 | 0.0843 | 1.400 – 1.810 |
| Aves | 1.580 | 0.0750 | 1.430 – 1.730 |
| Mammalia | 1.670 | 0.1158 | 1.400 – 1.940 |
| Amphibia *TimeDeath* | 0.0003 | 0.0030 | -0.006 – 0.006 |
| Reptilia *TimeDeath* | -0.0002 | 0.0015 | -0.003 – 0.003 |
| Aves *TimeDeath* | -0.012 | 0.0033 | -0.019 – -0.006 |
| Mammalia *TimeDeath* | -0.005 | 0.0028 | -0.01 – 0.0005 |
| Purity of DNA (log10) (N=586, R^2^ 0.02) | | | |
| *Linear model with mean purity per specimen* | | | |
| Amphibia | 0.216 | 0.0070 | 0.202 – 0.230 |
| Reptilia | 0.245 | 0.0053 | 0.235 – 0.256 |
| Aves | 0.243 | 0.0064 | 0.230 – 0.255 |
| Mammalia | 0.245 | 0.0074 | 0.230 – 0.260 |
| *TimeDeath* | -0.0003 | 0.0001 | -0.0005 – 0.00001 |
| *Linear mixed effect models* | | | |
| Amphibia | 0.227 | 0.0115 | 0.195 – 0.259 |
| Reptilia | 0.243 | 0.0104 | 0.216 – 0.270 |
| Aves | 0.240 | 0.0090 | 0.222 – 0.259 |
| Mammalia | 0.238 | 0.0139 | 0.204 – 0.272 |
| *TimeDeath* | -0.0002 | 0.0001 | -0.0005 – 0.00001 |

Linear regression results show how the amount and purity of DNA (log_10_ scale) were influenced by the estimated time of death (*TimeDeath*) and the taxonomic class (Class). Amount calculated as the mean value per specimen when multiple tissues were collected (Table 3 shows results when using the highest quantity obtained per specimen). Samples were collected during roadkill surveys in the Napo region of Ecuador from 2020 to 2021. We report estimated marginal means for each predictor and the interaction term (when interactions were significant), their standard error (SE), and their 95% confidence intervals (CI). For each model, we also report the number of specimens (*N*) for which data were available and the adjusted *R^2^* of the model.
