# Supplementary material for: From roads to biobanks: Roadkill animals as a valuable source of genetic data
Source: PLoS One. 2023 Dec 7;18(12):e0290836. doi: 10.1371/journal.pone.0290836 (PMC10703236; doi:10.1371/journal.pone.0290836)
Supplement: S2 Table — Samples were classified as “1” (minor degradation), “2” (medium degradation) o “3” (high degradation). (DOCX) [file pone.0290836.s004.docx]

**S2 Table. Qualitative assignment of DNA degradation.**

| Sample ID | Nucleic Acid (ng/µl) | Hours from death | Degradation level |
| --- | --- | --- | --- |
| 49 | 37.5 | 1 | 1 |
| 473 | 42.5 | 2 | 1 |
| 426 | 47.3 | 0 | 1 |
| 418 | 169.7 | 12 | 1 |
| 351 | 203.2 | 0 | 1 |
| 149 | 224.4 | 6 | 1 |
| 99 | 229.7 | 2 | 1 |
| 140 | 295.0 | 12 | 1 |
| 241 | 345.8 | 24 | 1 |
| 62 | 360.8 | 24 | 1 |
| 728 | 501.3 | 12 | 1 |
| 188 | 361.2 | 36 | 1 |
| 226 | 59.3 | 0 | 1 |
| 546 | 5.5 | 1 | 1 |
| 561 | 8.8 | 1 | 1 |
| 208 | 29.9 | 0 | 2 |
| 44 | 34.9 | 36 | 2 |
| 531 | 72.2 | 0.5 | 2 |
| 644 | 97.7 | 1 | 2 |
| 23 | 102.6 | 24 | 2 |
| 163 | 105.6 | 2 | 2 |
| 506 | 230.9 | 24 | 2 |
| 360 | 251.7 | 12 | 2 |
| 308 | 305.7 | 6 | 2 |
| 215 | 346.9 | 2 | 2 |
| 52 | 388.1 | 8 | 2 |
| 344 | 665.0 | 24 | 2 |
| 264 | 13.7 | 3 | 2 |
| 800 | 13.7 | 12 | 3 |
| 589 | 24.8 | 2 | 3 |
| 68 | 30.4 | 24 | 3 |
| 653 | 32.9 | 6 | 3 |
| 442 | 35.7 | 24 | 3 |
| 318 | 40.5 | 12 | 3 |
| 404 | 41.7 | 24 | 3 |
| 170 | 46.8 | 0 | 3 |
| 829 | 53.8 | 24 | 3 |
| 83 | 88.9 | 24 | 3 |
| 660 | 164.4 | 2 | 3 |
| 480 | 168.3 | 24 | 3 |
| 334 | 190.8 | 24 | 3 |
| 270 | 308.9 | 48 | 3 |
| 121 | 0.2 | 48 | 3 |
| 147 | 5.2 | 72 | 3 |
| 375 | 4.8 | 1 | 3 |
| 523 | 38.6 | 0 | 3 |
| 500 | 10.1 | 12 | 3 |
| 569 | 6.9 | 12 | 3 |
| 625 | 4.4 | 24 | 3 |
| 697 | 138.9 | 2 | 3 |
| 774 | 4.2 | 12 | 3 |
| 791 | 6.0 | 12 | 3 |
| 822 | 7.2 | 1 | 3 |

Samples were punctuated as “1” (minor degradation), “2” (medium degradation) o “3” according to its size of the smear for genomic DNA electrophoresis in samples isolated from intestine.
