# Supplementary material for: From roads to biobanks: Roadkill animals as a valuable source of genetic data
Source: PLoS One. 2023 Dec 7;18(12):e0290836. doi: 10.1371/journal.pone.0290836 (PMC10703236; doi:10.1371/journal.pone.0290836)
Supplement: Attachment — Submitted filename: PONE-D-23-09739.pdf [file pone.0290836.s007.pdf]

PLOS ONE

Roadkill animals as valuable source of genetic data to monitor the diversity of vertebrates and their associated microorganism without additional impact on wildlife

--Manuscript Draft--

**Manuscript Number:**	PONE-D-23-09739
**Article Type:**	Research Article
**Full Title:**	Roadkill animals as valuable source of genetic data to monitor the diversity of vertebrates and their associated microorganism without additional impact on wildlife
**Short Title:**	Roadkill animals as valuable source of genetic data
**Corresponding Author:**	Manuela Gonzalez-Suarez<br>University of Reading School of Biological Sciences<br>Reading, Berkshire UNITED KINGDOM
**Keywords:**	Roadkill, field samples, DNA stability, genetic resources, sequencing.
**Abstract:**	To protect biodiversity we must understand its structure and composition including the bacteria and microscopic parasites associated with wildlife, which may pose risks to human health. However, acquiring this knowledge often presents challenges particularly in areas of high diversity where there are many unknown species and funding resources can be limited. A solution to fill this knowledge gap is sampling roadkill (animals that die as a result of collisions with circulating vehicles). These specimens can help identify local wildlife and their associated parasites with fewer ethical and logistical challenges.<br>Here we test this approach analyzing 817 tissue samples from 590 roadkill vertebrate specimens (Amphibia, Reptilia, Aves and Mammalia) collected in roads within the Tropical Andes of Ecuador. First, we tested how the quantity and quality of recovered DNA varied across roadkill specimens collected at different times since death exploring if decomposition affected the potential to identify vertebrate species and associated microorganisms. Second, we compared DNA stability across taxa and tissues to identify potential limitations and offer recommendations for future work. Finally, we illustrate how these samples can aid in taxonomic identification and parasite detection.<br>Sampling roadkill can help study biodiversity. In our study DNA was recovered and amplified (allowing species identification and parasite detection) from roadkill even 120 hours after death, although risk of degradation increased overtime. DNA was extracted from all vertebrate classes but in smaller quantities and with lower quality from amphibians. We recommend sampling liver if possible as it produced the highest amounts of DNA (muscle produced the lowest). Additional testing of this approach in areas with different environmental and traffic conditions is needed, but our results show that sampling roadkill specimens could help detect and potentially monitor biodiversity and could be a valuable approach to create biobanks and preserve genetic data.
**Order of Authors:**	Manuel Alejandro Coba-Males
	Pablo Medrano-Vizcaíno
	Sandra Enríquez
	David Brito-Zapata
	Sarah Martin-Solano
	Sofía Ocaña-Mayorga
	Gabriel Alberto Carrillo-Bilbao
	Wilmer Narváez
	Jaime Antonio Salas
	Jazzmín Arrivillaga-Henríquez
	Manuela Gonzalez-Suarez
	Ana Poveda

Powered by Editorial Manager® and ProduXion Manager® from Aries Systems Corporation

Additional Information:	
**Question**	**Response**
**Financial Disclosure**<br><br>Enter a financial disclosure statement that describes the sources of funding for the work included in this submission. Review the submission guidelines for detailed requirements. View published research articles from *PLOS ONE* for specific examples.<br><br>This statement is required for submission and **will appear in the published article** if the submission is accepted. Please make sure it is accurate.<br><br>**Unfunded studies**<br>Enter: *The author(s) received no specific funding for this work.*<br><br>**Funded studies**<br>Enter a statement with the following details:<br>• Initials of the authors who received each award<br>• Grant numbers awarded to each author<br>• The full name of each funder<br>• URL of each funder website<br>• Did the sponsors or funders play any role in the study design, data collection and analysis, decision to publish, or preparation of the manuscript?<br>• **NO** - Include this sentence at the end of your statement: *The funders had no role in study design, data collection and analysis, decision to publish, or preparation of the manuscript.*<br>• **YES** - Specify the role(s) played.<br><br>* typeset	The authors would like to thank to: Corporación Ecuatoriana para el Desarrollo de la Investigación y Academia - CEDIA for the financial support given to the present research, development, and innovation work through its CEPRA program, especially for the Estudio de parásitos y microbioma de fauna silvestre en dos de las zonas más biodiversas del planeta: Los Andes Tropicales y Chocó-Darién en Ecuador fund (https://cedia.edu.ec/servicio/fondo-idi-universidades/cepra-xvi-2022/); Dirección de Investigación de la Universidad Central del Ecuador for Proyecto Senior 2021 fund (https://www.uce.edu.ec/web/di), and Reading University for SBS Seed Fund and a University International PhD studentship to PMV (https://www.reading.ac.uk/)
**Competing Interests**<br><br>Use the instructions below to enter a competing interest statement for this submission. On behalf of all authors, disclose any competing interests that could be perceived to bias this work—acknowledging all financial support and any other relevant financial or non-financial competing interests.	The authors have declared that no competing interests exist.

This statement is required for submission and **will appear in the published article** if the submission is accepted. Please make sure it is accurate and that any funding sources listed in your Funding Information later in the submission form are also declared in your Financial Disclosure statement.<br><br>View published research articles from *PLOS ONE* for specific examples.<br><br>**NO authors have competing interests**<br><br>Enter: *The authors have declared that no competing interests exist.*<br><br>**Authors with competing interests**<br><br>Enter competing interest details beginning with this statement:<br><br>*I have read the journal's policy and the authors of this manuscript have the following competing interests: [insert competing interests here]*<br><br>* typeset	
**Ethics Statement**<br><br>Enter an ethics statement for this submission. This statement is required if the study involved:<br><br>• Human participants<br>• Human specimens or tissue<br>• Vertebrate animals or cephalopods<br>• Vertebrate embryos or tissues<br>• Field research<br><br>Write "N/A" if the submission does not require an ethics statement.<br><br>General guidance is provided below. Consult the submission guidelines for detailed instructions. **Make sure that all information entered here is included in the**	MAAE-DBI-CM-2021-0215<br>MAAE-ARSFC-2021-1862<br>MAAE-ARSFC-2020-0791<br><br>Ministerio del Ambiente, Agua y Transición Ecológica from Ecuador (MAATE)

Powered by Editorial Manager® and ProduXion Manager® from Aries Systems Corporation

**Methods section of the manuscript.**<br><br>**Format for specific study types**<br><br>**Human Subject Research (involving human participants and/or tissue)**<br>• Give the name of the institutional review board or ethics committee that approved the study<br>• Include the approval number and/or a statement indicating approval of this research<br>• Indicate the form of consent obtained (written/oral) or the reason that consent was not obtained (e.g. the data were analyzed anonymously)<br><br>**Animal Research (involving vertebrate animals, embryos or tissues)**<br>• Provide the name of the Institutional Animal Care and Use Committee (IACUC) or other relevant ethics board that reviewed the study protocol, and indicate whether they approved this research or granted a formal waiver of ethical approval<br>• Include an approval number if one was obtained<br>• If the study involved *non-human primates*, add *additional details* about animal welfare and steps taken to ameliorate suffering<br>• If anesthesia, euthanasia, or any kind of animal sacrifice is part of the study, include briefly which substances and/or methods were applied<br><br>**Field Research**<br><br>Include the following details if this study involves the collection of plant, animal, or other materials from a natural setting:<br>• Field permit number<br>• Name of the institution or relevant body that granted permission	
**Data Availability**<br><br>Authors are required to make all data underlying the findings described fully available, without restriction, and from the time of publication. PLOS allows rare exceptions to address legal and ethical concerns. See the PLOS Data Policy and FAQ for detailed information.	Yes - all data are fully available without restriction

Powered by Editorial Manager® and ProduXion Manager® from Aries Systems Corporation

A Data Availability Statement describing where the data can be found is required at submission. Your answers to this question constitute the Data Availability Statement and **will be published in the article**, if accepted.<br><br>**Important:** Stating 'data available on request from the author' is not sufficient. If your data are only available upon request, select 'No' for the first question and explain your exceptional situation in the text box.<br><br>Do the authors confirm that all data underlying the findings described in their manuscript are fully available without restriction?	
**Describe where the data may be found in full sentences. If you are copying our sample text, replace any instances of XXX with the appropriate details.**<br><br>• If the data are **held or will be held in a public repository**, include URLs, accession numbers or DOIs. If this information will only be available after acceptance, indicate this by ticking the box below. For example: *All XXX files are available from the XXX database (accession number(s) XXX, XXX.*).<br>• If the data are all contained **within the manuscript and/or Supporting Information files**, enter the following: *All relevant data are within the manuscript and its Supporting Information files.*<br>• If neither of these applies but you are able to provide **details of access elsewhere**, with or without limitations, please do so. For example:<br><br>*Data cannot be shared publicly because of [XXX]. Data are available from the XXX Institutional Data Access / Ethics Committee (contact via XXX) for researchers who meet the criteria for access to confidential data.*<br><br>*The data underlying the results*	All relevant data are within the manuscript and its Supporting Information files.

presented in the study are available from (include the name of the third party and contact information or URL).<br>• This text is appropriate if the data are owned by a third party and authors do not have permission to share the data.<br><br>* typeset	
Additional data availability information:	Tick here if the URLs/accession numbers/DOIs will be available only after acceptance of the manuscript for publication so that we can ensure their inclusion before publication.

Powered by Editorial Manager® and ProduXion Manager® from Aries Systems Corporation

Manuscript Click here to access/download;Manuscript;Manuscript.docx

Roadkill animals as valuable source of genetic data to monitor the diversity of vertebrates and their associated microorganism without additional impact on wildlife

Manuel Alejandro Coba-Males[1¶], Pablo Medrano-Vizcaíno[2-3¶], Sandra Enríquez[1], David Brito-Zapata[4], Sarah Martin-Solano[5], Sofía Ocaña-Mayorga[6], Gabriel Alberto Carrillo-Bilbao[1], Wilmer Narváez[1], Jaime Antonio Salas[7-8], Jazzmín Arrivillaga-Henríquez[1], Manuela González-Suárez[2]*, Ana Poveda[1]*.

[1] Grupo de Investigación en Biodiversidad, Zoonosis y Salud Pública (GIBCIZ), Instituto de Salud Pública y Zoonosis (CIZ), Facultad de Ciencias Químicas (FCQ), Universidad Central del Ecuador, Quito, Ecuador.

[2]Ecology and Evolutionary Biology, School of Biological Sciences, University of Reading, Reading, United Kingdom.

[3]Universidad Regional Amazónica IKIAM, Grupo de Investigación Población y Ambiente, Tena, Ecuador.

[4]Instituto iBIOTROP, Museo de Zoología & Laboratorio de Zoología Terrestre, Universidad San Francisco de Quito USFQ, Quito, Ecuador.

[5]Grupo de Investigación en Sanidad Animal y Humana (GISAH), Carrera Ingeniería en Biotecnología, Departamento de Ciencias de la Vida y la Agricultura, Universidad de las Fuerzas Armadas—ESPE, Sangolquí, Ecuador.

[6]Centro de Investigación para la Salud en América Latina, Facultad de Ciencias Exactas y Naturales, Pontificia Universidad Católica del Ecuador, Quito, Ecuador.

[7]Facultad de Ciencias Naturales, Carrera de Biología, Universidad de Guayaquil, Ecuador.

[8]Facultad de Ingeniería, Universidad Espíritu Santo, Guayaquil, Ecuador.

* Corresponding authors

Email: manuela.gonzalez@reading.ac.uk (MGS); apoveda@uce.edu.ec (AP).

¶These authors contributed equally to this work.

Abstract

To protect biodiversity we must understand its structure and composition including the bacteria and microscopic parasites associated with wildlife, which may pose risks to human health. However, acquiring this knowledge often presents challenges particularly in areas of high diversity where there are many unknown species and funding resources can be limited. A solution to fill this knowledge gap is sampling roadkill (animals that die as a result of collisions with circulating vehicles). These specimens can help identify local wildlife and their associated parasites with fewer ethical and logistical challenges.

Here we test this approach analyzing 817 tissue samples from 590 roadkill vertebrate specimens (Amphibia, Reptilia, Aves and Mammalia) collected in roads within the Tropical Andes of Ecuador. First, we tested how the quantity and quality of recovered DNA varied across roadkill specimens collected at different times since death exploring if decomposition affected the potential to identify vertebrate species and associated microorganisms. Second, we compared DNA stability across taxa and tissues to identify potential limitations and offer recommendations for future work. Finally, we illustrate how these samples can aid in taxonomic identification and parasite detection.

Sampling roadkill can help study biodiversity. In our study DNA was recovered and amplified (allowing species identification and parasite detection) from roadkill even 120 hours after death, although risk of degradation increased overtime. DNA was extracted from all vertebrate classes but in smaller quantities and with lower quality from amphibians. We recommend sampling liver if possible as it produced the highest amounts of DNA (muscle produced the lowest). Additional testing of this approach in areas with different environmental and traffic conditions is needed, but our results show that sampling roadkill specimens could help detect and potentially monitor biodiversity and could be a valuable approach to create biobanks and preserve genetic data.

Introduction

Characterizing biodiversity including bacteria and microscopic parasites is key to ensure planetary and human health. However, acquiring this knowledge often presents challenges including ethical (the need to capture, handle, and take samples from living organisms without causing damage or death), logistical (the difficulty and cost of safely capturing and handling wildlife), and methodological (the challenges in identifying and quantifying biodiversity in less studied regions and of microscopic bacteria and parasites). In biodiverse, remote areas where funding and research infrastructure are more limited these challenges can be particularly strong. This knowledge gap could be partly reduced by capitalizing on a potential source of information: the animals that die on roads after collision with circulating vehicles (roadkill).

Human infrastructures including roads have important impacts on wildlife hindering movement (for food, mating, and refuge), and causing direct mortality [1]. These aspects can lead to population isolation, loss of genetic diversity, and in some cases

even increased extinction risk [2,3]. However, animals that die on roads can be valuable resources for research. Roadkill specimens can capture the (primarily vertebrate) biodiversity of a particular location and give information about species distributions, behavior, diet, and other ecological and physiological characteristics [4]. Unfortunately, the required taxonomical identification can be difficulty via direct examination due to the state of the specimen, which may be in various stages of decomposition or present damages because of the collision and in many cases from subsequent vehicles driving over. As an alternative collecting tissue from specimens could allow their identification via DNA sequencing [5].

Tissue collected from roadkill specimens can also provide very valuable information about associated microorganisms such as fungi, protozoan, bacteria, and viruses [6–10]. This information is important because these microorganisms can be pathogenic for the animal and are of interest to wildlife conservation. In addition, wildlife can act as reservoirs of human pathogens, becoming a health problem [9]. Roadkill sampling could be an interesting approach within the One Health framework that integrates animal, human, and environmental health [11]. Although, pathogen data can be obtained using traditional sampling methods (from live animals), these are often invasive and can negatively affect wildlife. Because of this, non-invasive sampling methods from excretions or feces are preferred; however, the amount of extracted DNA is lower [12,13]. There is a need for monitoring methods that are less invasive and impactful to wildlife [14] and sampling roadkill could be a useful approach in areas where roads exist. However, for such samples to be valuable we need to understand degradation and availability of DNA obtained from roadkill samples [15,16]. In particular we need to understand how the source of the sample (which taxa and tissue) and degree of

decomposition of the roadkill specimen affect DNA quantity and quality (purity and integrity).

Here we address these questions analyzing 817 samples from 590 roadkill specimens representing amphibians, reptiles, birds and mammals found dead on roads of the Napo province in Ecuador. Whenever possible we collected samples from multiple tissues in the same individual. In addition to reporting on DNA quantity and quality outputs we also tested the value of the genetic samples for: genetic identification of specimens that cannot be identified from direct observation (eg, highly damaged specimens), and detection of microorganisms within wildlife, in particular of the protozoan *Leishmania* spp. These parasites have a wide distribution in humans and can parasite domestic animals like dogs and cats [17], wild mammals [18,19], and likely birds and reptiles [19–21].

Methods

Study area and roadkill sampling

This work was conducted in the Amazonian province of Napo in Ecuador, across an area surrounded by four protected areas: Antisana Ecological Reserve, Sumaco-Napo-Galeras National Park, Cayambe-Coca National Park, and Colonso Chalupas Biological Reserve (Fig 1). The annual average temperature ranges from 4.63 to 23.7°C, and the annual precipitation from 1100 to 3400 mm. The study area is described in more detail in previous publication [22,23].

During 100 non-consecutive days from the 19th of September 2020 to the 23rd of March 2021, we monitored 240 km of primary and secondary roads using a car at an average velocity of 40 km/h. Each time we found a carcass, we stopped the car, collected the whole individual or muscle samples (if the state of the carcass did not allow whole collection and subsequent organ extraction), and stored them in a container with dry ice. Collected individuals were taken to the laboratory to obtain samples of muscle, spleen, heart, liver, lung, large intestine, and brain. The condition of some specimens did not allow for obtaining samples of all tissues. Samples were frozen at -80°C until their processing for DNA extraction.

For each specimen we estimated the time of death, *TimeDeath*, in time blocks representing: 0, 12, 24, 36, 48, 60, 72, 96, or 120 hours. The ability to estimate these stages had been developed during previous studies in which roadkill specimens were marked and revisited carcasses daily for ten days to characterize degradation [22,24].

DNA extraction and evaluation of quantity and quality

We processed all the samples to extract DNA from tissues with a commercial kit (PureLink™ Genomic DNA Mini Kit, from Invitrogen by Thermo Fisher Scientific) following the manufacturer’s instructions. We measured the quantity of nucleic acids using a Nanodrop 2000 (Thermo Fisher Scientific) with values transformed into ng/µL (the values of DNA quantification obtained are in the S1 File). To assess DNA quality we considered two parameters: the purity given by the ratio A260/A280, and integrity. Integrity was evaluated in all collected samples of intestine tissue (as we were interested in microbiome and gastrointestinal parasites) and qualitatively classified depending on the size of the smear observed in gel electrophoresis [25]. The classification was as follow: “minor degradation” (sizes over 6000 pb), “medium degradation” (sizes from

1500 pb to 6000 pb), or “high degradation” (sizes below 1500 pb or not visualized DNA).

Statistical analyses

We tested if DNA quantity and purity were influenced by estimated *TimeDeath* and the taxonomic class of the roadkill specimen using linear regression models with the function lm in base R [26]. We fitted models with only additive terms as well as models with interaction terms. We report the most parsimonious model (if the interaction term was not significant, we report results from the additive model). To avoid pseudoreplication caused by including data from multiple tissues obtained from the same specimen, we selected the sub-sample with higher DNA concentration (ng/µL). Results were qualitatively the same if we instead used the mean value calculated across tissues from the same specimen (S1 Table). In addition, to test the robustness of results to the potential non-independence of data obtained from related organisms from the same taxa and thus, shared evolutionary history, we also fitted linear mixed effects regression models using the function lmer from the lme4 package [27] in R with taxonomic order and family as nested values random effects. Because not all specimens could be identified to order or family, mixed effect models included fewer specimens.

To test if DNA quantity and purity were influenced by the sampled tissue, we focus on tissues sampled from at least 50 specimens (obtaining samples from some tissues were difficult due to their size and degradation). We analyzed all samples, including replicates from the same specimen, and thus to avoid pseudoreplication we fitted mixed effects regression models with specimen ID as a random factor using the function lmer from the lme4 package in R.

To meet assumptions of normality and homoscedasticity of residuals we $\log_{10}$ transformed DNA quantity and quality values. Model assumptions (linearity, normality and homoscedasticity of residuals, and lack of outliers) were visually checked using the function check_model from the performance package [28] in R. This function also returns Variance Inflation Factors (VIF) that were used to test for collinearity among predictors. We used the function emtrends from the package emmeans [29] to estimate the estimated marginal means reported as model output.

Molecular identification of vertebrates and parasites

For identification of vertebrate specimens we analyzed mitochondrial *cytochrome C oxidase subunit 1* (*COI*) with 10 pmol/µL of two cocktails of primers: cocktail forward (C_VF1LFt1) contains VF1_t1: VF1d_t1: LepF1_t1:VF1i_t1 (1:1:1:3) and cocktail reverse (C_VR1LRt1) contains VR1_t1: VR1d_t1: LepRI_t1: VR1i_t1 (1:1:1:3) (Table 1). Analyses focused on eight specimens (seven reptiles and one amphibian) that could not be identified based on morphological features only as our goal was to generate a complete identified dataset.

Table 1. PCR primers used in this study to test potential of genetic identification of vertebrate roadkill specimens and *Leishmania* spp.

COI		
Name	Primer sequence 5’-3’	Reference
LepF1_t1	TGTAAAACGACGGCCAGTATTCAACCAATCATAAAGATATTGG	[30]
VF1_t1	TGTAAAACGACGGCCAGTTCTCAACCAACCACAAAGACATTGG	
VF1d_t1	TGTAAAACGACGGCCAGT TCTCAACCAACCACAARGAYATYGG	
VF1i_t1	TGTAAAACGACGGCCAGTTCTCAACCAACCAIAAIGAIATIGG	
LepR1_t1	TGTAAAACGACGGCCAGTATTCAACCAATCATAAAGATATTGG	
VR1_t1	TGTAAAACGACGGCCAGTTCTCAACCAACCACAAAGACATTGG	
VR1d_t1	TGTAAAACGACGGCCAGT TCTCAACCAACCACAARGAYATYGG	
VR1i_t1	TGTAAAACGACGGCCAGTTCTCAACCAACCAIAAIGAIATIGG	

18S SSU rRNA		
Name	Primer sequence 5’-3’	Reference
SLF	GCTTGTTTCAAGGACTTAGC	[31]
S762R	GACTTTTGCTTCCTCTAATG	
S823F	CGAACAACTGCCCTATCAGC	
S662R	GACTACAATGGTCTCTAATC	
S825F	ACCGTTTCGGCTTTTGTTGG	
SLIR	ACATTGTAGTGCGCGTGTC	

The polymerase chain reaction (PCR) was performed with Platinum™ Taq DNA Polymerase from Invitrogen as described by Hebert [32] and colleagues with some modifications; 2.5 µL buffer 10 X, 1.15 µL (2.3 mM) MgCl2, 0.12 µL (0.046 mM) dNTPs, 0.25 µL (0.1 mM) each cocktail of oligonucleotides (C_VF1LFt1 and C_VR1LRt1), 7.5 µL (0.3 mg/mL) of BSA, 0.5 U of Taq polymerase and 100 ng of DNA for reaction in a final volume of 25 µL. PCR amplification was performed with an initial denaturation step (94°C, 1 min), followed by 5 cycles of denaturation (94˚C, 1 min), annealing (50˚C, 40 sec), and polymerization (72˚C, 1 min), followed of 30 cycles of denaturation (95˚C, 15 sec), annealing (54˚C, 20 sec) and polymerization (72˚C, 45 sec), and a final step of 10 min at 72 °C.

To detect *Leishmania* spp. we amplified a small subunit (18S) of ribosomal RNA (*18S SSU-rRNA*) from liver samples of roadkill specimens [33] using universal primers (Table 1). We first used the external primers SLF/S762R (primary amplification) and the internal primers S825F/SLIR targeting a ~959 bp product of the first half (Nt) of the *18S SSU-rRNA* locus [31]. The polymerase chain reaction (PCR) was performed with Platinum™ Taq DNA Polymerase from Invitrogen with 2.5 µL buffer 10 X, 0.75 µL (1.5 mM) MgCl2, 0.5 µL (0.2 mM) dNTPs, 1 µL (0.4 µM) each oligonucleotide, 0.5 U of Taq polymerase and 100 ng of DNA for reaction in a final volume of 25 µL. PCR amplification was as described previously for primary and secondary *18S SSU-rRNA* [31].

We visualized the PCR products by DNA electrophoresis on a 1.5% agarose gel. Positive amplicons, identified by their size (~900 bp for the *COI* gene and 959 bp for the *18 SSU-rRNA* gene), were sequenced in two directions, forward and reverse (Sanger method) by Macrogen (South Korea). Resulting sequences were trimmed and edited with Bioedit and MEGA 11 software [34,35] to perform pairwise alignment, assembly the contig and create consensus sequences which were then analyzed by BLAST using the blastn algorithm in NCBI (National Center for Biotechnology Information) [36]. To assign sequences to *Leishmania* spp. or relevant vertebrate genera we used a threshold for the identity of 85 % and of 90% for cover.

Results

We obtained 817 samples representing 590 unique roadkill specimens (Table 2, S1 File). From 516 specimens we collected samples from a single tissue; the other 74 specimens were represented by samples from 2-7 different tissues.

Nucleic acids were successfully extracted from 812 samples representing 586 unique roadkill specimens with time of death varying between 0 and 120 hours. For four individuals with single tissue samples, no DNA was obtained after extraction. In addition, no DNA was obtained from one brain sample from one specimen but extractions were successful for five other tissues obtained from that individual. Table 2 summarizes the samples analysed.

Table 2. Samples analyzed in this study.

	**Muscle**	**Brain**	**Liver**	**Heart**	**Intestine**	**Blood**	**Blood vessel**	**Bone**	**Lung**	**Skin**	**Spleen**

**Mammalia**	100	1	4	3	2	0	2	2	4	2	2	122
**Aves**	114	21	19	26	21	3	5	0	19	2	0	230
**Amphibia**	116	1	3	2	3	0	6	0	1	0	0	132
**Reptilia**	136	25	31	26	27	0	57	5	15	4	2	328
**TOTAL**	466	48	57	57	53	3	70	7	39	8	4	**812**

Total number of samples collected from different tissues in roadkill specimens representing distinct taxonomic classes and analyzed in this study.

Effect of time of death on DNA quantity and purity

We obtained DNA from specimens representing all sampled taxonomic classes and from specimens estimated to have died 120 hours before collection (the oldest analyzed here). The four specimens from which no DNA was obtained were estimated to have died between 12 and 72 hours.

DNA quantity and purity were similar among taxonomic groups except for Amphibia specimens, which returned significantly lower amounts and less pure DNA (Fig 2, Table 3).

DNA quantity was significantly and negatively associated with the time the specimen had been on the road after collision in birds but not in other taxonomic classes (Fig 3, Table 3). DNA purity was not affected by estimated time since death with no significant interaction (Table 3).

Table 3. **Linear regression models testing how DNA quantity and purity varied by class and time since death.**

Predictor	Marginal means	SE	95% CI
Quantity of DNA (log10) (N=586, R^2 0.18)			
**Amphibia**	1.010	0.0544	0.906 – 1.120
**Reptilia**	1.610	0.0408	1.531 – 1.690
**Aves**	1.640	0.0494	1.542 – 1.740
**Mammalia**	1.720	0.0574	1.604 – 1.830
**Amphibia *TimeDeath***	-0.002	0.0028	-0.0072 – 0.0038
**Reptilia *TimeDeath***	-0.001	0.0015	-0.0031 – 0.00263
**Aves *TimeDeath***	-0.009	0.0023	-0.0136 – -0.0047
**Mammalia *TimeDeath***	-0.004	0.0023	-0.0081 – 0.00091
Purity of DNA (log10) (N=586, R^2 0.02)			
**Amphibia**	0.217	0.0067	0.204 – 0.230
**Reptilia**	0.245	0.0050	0.235 – 0.255
**Aves**	0.243	0.0061	0.231 – 0.255
**Mammalia**	0.245	0.0070	0.231 – 0.259
TimeDeath	-0.0002	0.0001	-0.0005 – 0.00001

Linear regression results show how the amount and purity of DNA (log_{10} scale) were influenced by the estimated time of death (*TimeDeath*) and the taxonomic class (Class). Samples were collected during roadkill surveys in the Napo region of Ecuador from 2020 to 2021. We report estimated marginal means for each predictor and the interaction term (when interactions were significant), their standard error (SE), and their 95% confidence intervals (CI). For each model, we also report the number of specimens (*N*) for which data were available and the adjusted R^2 of the model. Results were qualitatively the same when accounting for evolutionary relationships in random effects models and when considering the mean of DNA amount per specimen (when multiple tissues were analysed) instead of the best value (S1 Table).

To identify the type of tissue that had an effect on DNA quantity or purity we performed the analysis with tissues represented at least by 50 samples or more. We collected 703 samples from five different tissues: muscle (n = 466), blood vessels (n = 70), heart (n = 57), liver (n = 57), and intestine (n = 53); (see Table 2). DNA quantity varied among the compared tissues, with liver returning significantly more and muscle generally less (Fig 4, Table 4). There were no differences in DNA purity among tissues (Table 4).

Table 4. **Linear regression of DNA quantity by tissue.**

Predictor	Marginal means	SE	95% CI
Quantity of DNA (log10) (N=703, conditional R^2 0.51, marginal R^2 0.21)			
**Amphibia**	1.27	0.066	1.14 – 1.40
**Reptilia**	1.70	0.045	1.61 – 1.78
**Aves**	1.80	0.054	1.69 – 1.90
**Mammalia**	1.92	0.069	1.79 – 2.06
**Blood vessel**	1.58	0.070	1.44 – 1.72
**Heart**	1.66	0.075	1.51 – 1.81
**Intestine**	1.72	0.077	1.56 – 1.87
**Liver**	2.00*	0.075	1.86 – 2.15
**Muscle**	1.39*	0.027	1.34 – 1.44
Purity of DNA* (log10) *(N=703, conditional R^2 0.10, marginal R^2 0.03)			
**Amphibia**	0.22	0.009	0.204 – 0.240
**Reptilia**	0.25	0.006	0.235 – 0.259
**Aves**	0.24	0.007	0.229 – 0.257
**Mammalia**	0.25	0.010	0.228 – 0.267

**Blood vessel**	0.24	0.011	0.222 – 0.265
**Heart**	0.27	0.012	0.242 – 0.288
**Intestine**	0.22	0.012	0.192 – 0.240
**Liver**	0.24	0.012	0.217 – 0.263
**Muscle**	0.24	0.004	0.227 – 0.243

Linear mixed effect regression results show how the amount and purity of DNA ($\log_{10}$ scale) extracted from roadkilled specimens were influenced by the analyzed tissue and taxonomic class (Class). Samples were collected during roadkill surveys in the Napo region of Ecuador completed in 2020-2021. We report estimated marginal means for each predictor and the interaction term (when interactions were significant), and their 95% confidence intervals (CI). For each model, we also report the number of specimens (N) for which data were available and the adjusted R^2 of the model.

DNA integrity

The 53 intestine samples contained a DNA quantity ranging from 0.2 to 665 ng/µL, but we observed a tendency (not significant) for DNA to be more degraded in samples with longer times since death (Fig 5A), probably due to exposure to environmental conditions such as rain or high temperatures. Samples scored as high level of degradation had significantly lower quantities of DNA (Fig 5B).

Successful amplification of specific targets by PCR

To assess the use DNA for genetic identification of species, we selected eight samples from reptiles and amphibian which taxonomical identification was not confirmed due to

the decaying state of the individual. The selected samples were used to amplify a region of 900 pb of *cytochrome C oxidase subunit 1* (*COI*). Amplification was successful in all the samples (Fig 6B) despite the variability in the integrity of the extracted genomic DNA (Fig 6A. Table 5). We obtained cover values >95%, but the identity values were lower, potentially because there may be no matching sequences uploaded to the NCBI repository. However, while more analyses should be done before making conclusion about these specimens, we considered values of identity ≥ 85 % are indicative of genus (Table 6), as corroborated by taxonomical identification.

Table 5. Qualitative assignment of DNA degradation from vertebrate samples

Sample ID	391	464	465	467	468	682	683	684
**DNA (ng/µl)**	11.7	17.7	230.6	23.8	10.8	112.0	15.2	191.0
**A260/A280**	1.82	1.98	1.86	1.79	1.67	1.81	1.72	1.82
**Degradation level**	3	2	1	2	1	2	3	2
**Hours from death**	24	0	12	24	0	36	24	24

The concentration (ng/µL), the purity (A260/280), the degradation level of DNA and the hours of death, assigned to each sample used for molecular identification of vertebrate species in Fig 6.

Table 6. Results from local alignment of *COI* sequences amplified from seven reptiles and one amphibian using BLAST

ID	Class	Length (pb)	Cover	Identity	Accession N°	Reference	Size (bp)
**391**	Reptilia	664	99	87,18	AB079597.1	*Rena humilis* mitochondrial DNA	1548
**464**	Reptilia	671	97	93,12	MH140069.1	*Atractus imperfectus* voucher CH:9399 *cytochrome oxidase subunit 1* (*COI*) gene, partial.	671
**465**	Reptilia	656	98	92,39	MH140069.1	*Atractus imperfectus* voucher CH:9399 *cytochrome oxidase subunit 1* (*COI*) gene, partial.	654
**467**	Reptilia	656	99	89,08	MH140069.1	*Atractus imperfectus* voucher CH:9399	654

						cytochrome oxidase subunit 1 (*COI*) gene, partial.	
**468**	Amphibia	670	100	84,33	KF540146.1	*Caecilia tentaculata* mitochondrion	1553
**682**	Reptilia	661	97	92,37	MH140069.1	*Atractus imperfectus* voucher CH:9399 *cytochrome oxidase subunit 1* (*COI*) gene, partial.	654
**683**	Reptilia	663	98	93,24	MH140069.1	*Atractus imperfectus* voucher CH:9399 *cytochrome oxidase subunit 1* (*COI*) gene, partial.	654
**684**	Reptilia	661	97	92,52	MH140069.1	*Atractus imperfectus* voucher CH:9399 *cytochrome oxidase subunit 1* (*COI*) gene, partial.	654

Amplification was successful for *Leishmania* spp. in 9 of the 57 liver samples (Fig. 7; Table [illegible]). With cover ≥ 98 % and identity ≥ 99 % values we had high certainty in the match to genus and likely to the species *L. amazonensis* in concordance with previous records from Ecuador [37–39].

While additional testing of the potential for using roadkill species for taxonomic identification and parasitology our results are promising. We were able to amplify gene fragments from DNA samples with concentration as low as 7.1 ng/µL (sample 209, Fig 7, Table 7), and with high degradation (samples 391 and 683, Fig 6; samples 209 and 819, Fig 7).

Table 7. Qualitative assignment of DNA degradation from liver samples

Sample ID	209	218	466	499	652	775	788	819	831
**DNA (ng/µl)**	7.1	188.5	11.7	35.9	125.9	123.8	55.3	38.1	261.2
**A260/A280**	1.52	1.85	1.87	1.66	1.71	1.80	1.76	1.68	1.77
**Degradation level**	3	2	1	1	3	2	2	3	2
**Hours from death**	0	24	6	0	6	12	12	1	24

The concentration (ng/µL), the purity (A260/280), the degradation level of DNA and the hours of death, assigned to each sample used for molecular identification of *Leishmania* spp. in Fig 7.

Table 8. Results from local alignment of *18S SSU-rRNA* sequences amplified from liver samples using BLAST

ID	Length (pb)	Cover	Identity	Accession N°	Reference	Size (bp)
**209**	915	99	100	GQ332354.1		
**218**	908	99	99,89	GQ332354.1		
**466**	925	98	100	GQ332354.1		
**499**	919	99	100	GQ332354.1	*Leishmania amazonensis* 18S ribosomal RNA gene, complete sequence; and internal transcribed spacer 1, partial sequence	2191
**652**	908	99	99,89	GQ332354.1		
**775**	911	99	100	GQ332354.1		
**788**	914	99	100	GQ332354.1		
**819**	913	99	100	GQ332354.1		
**831**	914	99	99,89	GQ332354.1		

Discussion

We show here that tissues samples collected from roadkill wildlife can offer a source of genetic data that can contribute to our understanding of biodiversity patterns, providing information to identify vertebrates and allowing the detection and potential monitoring of microorganisms including those that can pose risks to human health. While the

necessary genetic analyses can be costly (even if becoming more and more affordable), sampling roadkill is a simple (one can drive to collect samples), low cost, low risk and low impact (not capturing of wildlife) to gather tissue from wildlife. While not all wildlife crosses roads or become roadkill, we know maybe species are affected [40–42] and thus, this approach can provide access to a diversity of species. The resulting data could contribute to gain much needed understanding of biodiversity in less-studied areas and be used in combination to other approaches such as environmental DNA (eDNA) and metabarcoding [43,44].

In our study region, a tropical environment with relatively high humidity and non-extreme temperatures (from 4.63 to 23.7°C), we were able to extract DNA from nearly all samples (DNA was not obtained only from five, <1% of samples), even from specimens estimated to have been on the road for 120 hours (5 days). Suggesting this method could be implemented without the need to sample daily or too frequently to capture available samples, and with high conversion of tissue collected to genetic material. However, the potential of obtain good quality and pure DNA may vary across regions depending on environmental conditions, mainly temperature and humidity, but also the acidity of the soil, and UV exposition [45]; as well as the levels of traffic, which can lead to quick degradation of roadkill specimens.

DNA was obtained from specimens in all four sampled vertebrate groups but we obtained less and lower quality DNA from amphibian samples. While we are not sure of why this occur it is possible that the highly permeable dermis of amphibians [46,47] could affect sample quality, by allowing substances present in the environment, potential detrimental to DNA, to penetrate amphibian tissues and cells. In addition, we also found that bird samples produced lower quantities of DNA when collected in older

specimens (longer times since death), while this trend was not detected in other taxonomic groups. We are not sure why bird samples may be more affected by time, but it would be interesting to explore if this pattern is also observed in other regions.

DNA was also obtained from all types of tissues samples, but liver samples produced higher DNA quantities, and muscle generally less. We did not find significant literature helping us to understand the yield of DNA we obtain from different tissues, but in few previous works authors get also higher DNA concentrations from liver [48,49]. It is know that regenerating hepatocytes are tetraploids [50]. This and other aspects, such as cell density but also, how environmental conditions may affect different organs and tissues [45] should be explored deeply for a better understanding.

Not surprisingly, DNA degraded over time (since death). DNA fragmentation can interfere amplification of complete target genes and result in sequencing errors [51]. However, we found that observed levels of degradation did not affect amplification for vertebrate identification. However, it may have affected our ability to amplify parasite DNA which would be found in much lower quantities within the host tissue leading to a difficult or inefficient amplification [52]. We used a nested PCR technique with high sensibility and specificity for diagnostic of visceral leishmaniasis for samples with low parasitaemia [53]. We were able to amplify DNA from *Leishmania* spp. parasites in several samples, whether parasites had in fact been present in other specimens but we could not detect them is unclear.

Overall, the data presented here indicates that DNA extracted from carcasses of roadkill animals can have enough quality for downstream applications. Our findings are consistent with conclusions from studies considering postmortem DNA degradation in forensic samples, for example to identify missing people or sequence museum samples [5,25]. Biobanks of tissue and/or DNA can be generated from samples collected from

roadkill animals without adding an impact on wildlife, and help answer questions about species distributions, diseases, taxonomy and generally contribute to preserve biological information. We are not suggesting roads should be viewed as beneficial because of the potential of collecting samples, it is important to reduce the impact of infrastructure on wildlife. However, roads are also necessary and roadkills cannot be fully avoided. Here we suggest these carcases are collected and recognized as precious samples that can help us draw a fuller picture of local biodiversity, especially in countries as Ecuador with high rates of diversity and endemism.

Acknowledgments

We thank F. Vaca, R. Rodríguez and V. Herrera from Universidad Central del Ecuador and X. Fiallos from Yachay, who helped us in implementing this project. We thank also to Ministerio del Ambiente, Agua y Transición Ecológica from Ecuador (MAATE), for the concession of legal permissions for samples collection: Estudio e identificación molecular de parásitos y microbioma presentes en fauna silvestre del Ecuador, No. MAAE-DBI-CM-2021-0215, MAAE-ARSFC-2021-1862 and MAAE-ARSFC-2020-0791.

References

1. Bischof R, Steyaert SMJG, Kindberg J. Caught in the mesh: roads and their network-scale impediment to animal movement. Ecography (Cop). 2017;40: 1369–1380. doi:https://doi.org/10.1111/ecog.02801

2. Grilo C, Borda-de-Água L, Beja P, Goolsby E, Soanes K, le Roux A, et al.

Conservation threats from roadkill in the global road network. Glob Ecol Biogeogr. 2021;30: 2200–2210. doi:https://doi.org/10.1111/geb.13375

3. Vaeokhaw S, Ngoprasert D, Swatdipong A, Gale GA, Klinsawat W, Vichitsoonthonkul T. Effects of a highway on the genetic diversity of Asiatic black bears. Ursus. 2020;2020: 1–15. doi:10.2192/URSUS-D-18-00013.2

4. Schwartz ALW, Shilling FM, Perkins SE. The value of monitoring wildlife roadkill. Eur J Wildl Res. 2020;66. doi:10.1007/s10344-019-1357-4

5. Allio R, Tilak MK, Scornavacca C, Avenant NL, Kitchener AC, Corre E, et al. High-quality carnivoran genomes from roadkill samples enable comparative species delineation in aardwolf and bat-eared fox. Elife. 2021;10: 1–77. doi:10.7554/eLife.63167

6. Motley JL, Stamps BW, Mitchell CA, Thompson AT, Cross J, You J, et al. Opportunistic Sampling of Roadkill as an Entry Point to Accessing Natural Products Assembled by Bacteria Associated with Non-anthropoidal Mammalian Microbiomes. J Nat Prod. 2017;80: 598–608. doi:10.1021/acs.jnatprod.6b00772

7. Pendl H, Hernández-Lara C, Kubacki J, Borel N, Albini S, Valkiūnas G. Exo-erythrocytic development of Plasmodium matutinum (lineage pLINN1) in a naturally infected roadkill fieldfare Turdus pilaris. Malar J. 2022;21: 1–13. doi:10.1186/s12936-022-04166-x

8. Campbell SJ, Ashley W, Gil-Fernandez M, Newsome TM, Di Giallonardo F, Ortiz-Baez AS, et al. Red fox viromes in urban and rural landscapes. Virus Evol. 2020;6: 1–11. doi:10.1093/ve/veaa065

9. Szekeres S, Docters van Leeuwen A, Tóth E, Majoros G, Sprong H, Földvári G.

Road-killed mammals provide insight into tick-borne bacterial pathogen communities within urban habitats. Transbound Emerg Dis. 2019;66: 277–286. doi:10.1111/tbed.13019

10. Arrivillaga J, Caraballo V. Medicina de la Conservación. Rev Biomédica. 2009;20: 55–67. doi:10.32776/revbiomed.v20i1.528

11. Destoumieux-Garzón D, Mavingui P, Boetsch G, Boissier J, Darriet F, Duboz P, et al. The one health concept: 10 years old and a long road ahead. Front Vet Sci. 2018;5: 1–13. doi:10.3389/fvets.2018.00014

12. Chaves PB, Paes MF, Mendes SL, Strier KB, Louro ID, Fagundes V. Noninvasive genetic sampling of endangered muriqui (Primates, Atelidae): Efficiency of fecal DNA extraction. Genet Mol Biol. 2006;29: 750–754. doi:10.1590/S1415-47572006000400028

13. Carrillo-Bilbao G, Navarro JC, Martin-Solano S, Chávez-Larrea MA, Cholota-Iza C, Saegerman C. First Molecular Identification of Trypanosomes and Absence of Babesia sp. DNA in Faeces of Non-Human Primates in the Ecuadorian Amazon. Pathogens. 2022;11: 1–20. doi:10.3390/pathogens11121490

14. Schilling AK, Mazzamuto MV, Romeo C. A Review of Non-Invasive Sampling in Wildlife Disease and Health Research: What’s New? Animals. 2022;12: 1–24. doi:10.3390/ani12131719

15. Kuhn R, Böllmann J, Krahl K, Bryant IM, Martienssen M. Data on DNA gel sample load, gel electrophoresis, PCR and cost analysis. Data Br. 2018;16: 732–751. doi:10.1016/j.dib.2017.11.082

16. Kuhn R, Böllmann J, Krahl K, Bryant IM, Martienssen M. Comparison of ten

different DNA extraction procedures with respect to their suitability for environmental samples. J Microbiol Methods. 2017;143: 78–86. doi:10.1016/j.mimet.2017.10.007

17. Cardoso RM, De Araújo NNSL, Sierra Romero GA, Minuzzi Souza TTC, Dietrich AG, Mendes JD, et al. Expanding the knowledge about Leishmania species in wild mammals and dogs in the Brazilian savannah. Parasites and Vectors. 2015;8: 1–8. doi:10.1186/s13071-015-0780-y

18. Azami- conesa I, Gómez- muñoz MT, Martínez- díaz RA. A systematic review (1990–2021) of wild animals infected with zoonotic leishmania. Microorganisms. 2021;9. doi:10.3390/microorganisms9051101

19. Cardoso L, Schallig H, Persichetti MF, Pennisi MG. New epidemiological aspects of animal leishmaniosis in europe: The role of vertebrate hosts other than dogs. Pathogens. 2021;10. doi:10.3390/pathogens10030307

20. Chen H, Li J, Zhang J, Guo X, Liu J, He J, et al. Multi-locus characterization and phylogenetic inference of Leishmania spp. In snakes from Northwest China. PLoS One. 2019;14. doi:10.1371/journal.pone.0210681

21. Mendoza-Roldan JA, Latrofa MS, Tarallo VD, Manoj RRS, Bezerra-Santos MA, Annoscia G, et al. Leishmania spp. in Squamata reptiles from the Mediterranean basin. Transbound Emerg Dis. 2022;69: 2856–2866. doi:https://doi.org/10.1111/tbed.14438

22. Medrano-Vizcaíno P, Espinosa S. Geography of roadkills within the Tropical Andes Biodiversity Hotspot: Poorly known vertebrates are part of the toll. Biotropica. 2021;53: 820–830. doi:10.1111/btp.12938

23. Medrano-Vizcaíno P, Grilo C, Brito-zapata D, González-Suárez M. The road of > 1000 corpses : landscape and road- related features that promote mortality in the Amazon. Prepr (Version 1) available Res Sq. 2022; 1–18. doi:10.21203/rs.3.rs-2156016/v1

24. Tozzo P, Scrivano S, Sanavio M, Caenazzo L. The role of DNA degradation in the estimation of post-mortem interval: A systematic review of the current literature. Int J Mol Sci. 2020;21: 3540. doi:10.3390/ijms21103540

25. Stotesbury T, Cossette ML, Newell-Bell T, Shafer ABA. An Exploratory Time Since Deposition Analysis of Whole Blood Using Metrics of DNA Degradation and Visible Absorbance Spectroscopy. 2021;178: 735–743. doi:10.1007/s00024-020-02494-0

26. R Core Team. R: A language and environment for statistical computing. R Foundation for Statistical Computing. Vienna, Austria.: R Foundation for Statistical Computing; 2021. Available: https://www.r-project.org/.

27. Bates D, Mächler M, Bolker B, Walker S. Fitting Linear Mixed-Effects Models Using lme4. J Stat Softw. 2015;67: 1–48. doi:10.18637/jss.v067.i01

28. Lüdecke D, Ben-Shachar MS, Patil I, Waggoner P, Makowski D. performance: An R Package for Assessment, Comparison and Testing of Statistical Models. J Open Source Softw. 2021;6: 3139. doi:https://doi.org/10.21105/joss.03139

29. Lenth R V. emmeans: Estimated Marginal Means, aka Least-Squares Means. 2022.

30. Ivanova N V, Zemlak TS, Hanner RH, Hebert PDN. Universal primer cocktails for fish DNA barcoding. Mol Ecol Notes. 2007;7: 544–548. doi:10.1111/j.1471-

8286.2007.01748.x

31. Egan SL, Ruiz-Aravena M, Austen JM, Barton X, Comte S, Hamilton DG, et al. Blood parasites in endangered wildlife-trypanosomes discovered during a survey of haemoprotozoa from the tasmanian devil. Pathogens. 2020;9: 1–18. doi:10.3390/pathogens9110873

32. Clare EL, Lim BK, Engstrom MD, Eger JL, Hebert PDN. DNA barcoding of Neotropical bats: Species identification and discovery within Guyana: Barcoding. Mol Ecol Notes. 2007;7: 184–190. doi:10.1111/j.1471-8286.2006.01657.x

33. Modabberi F, Ghadimi SN, Shahriarirad R, Nadimi E, Karbalay-doust S, Rashidi S, et al. Stereological analysis of liver, spleen and bone of Leishmania infantum-experimentally infected hamsters. Exp Parasitol. 2021;228: 108137. doi:https://doi.org/10.1016/j.exppara.2021.108137

34. Hall TA. BioEdit: A User-Friendly Biological Sequence Alignment Editor and Analysis Program for Windows 95/98/NT. Nucleic Acids Symp Ser. 1999;41: 95–98.

35. Tamura K, Stecher G, Kumar S. MEGA11: Molecular Evolutionary Genetics Analysis Version 11. Mol Biol Evol. 2021;38: 3022–3027. doi:10.1093/molbev/msab120

36. Sayers EW, Bolton EE, Brister JR, Canese K, Chan J, Comeau DC, et al. Database resources of the national center for biotechnology information. Nucleic Acids Res. 2022;50: D20–D26. doi:10.1093/nar/gkab1112

37. Arrivillaga-Henríquez J, Enríquez S, Romero V, Echeverría G, Pérez-Barrera J, Poveda A, et al. Eco-epidemiological aspects, natural detection and molecular

identification of Leishmania spp. in Lutzomyia reburra, Lutzomyia barrettoi majuscula and Lutzomyia trapidoi. Biomedica. 2017;37.

38. Kato H, Gomez EA, Martini-Robles L, Muzzio J, Velez L, Calvopiña M, et al. Geographic Distribution of Leishmania Species in Ecuador Based on the Cytochrome B Gene Sequence Analysis. PLoS Negl Trop Dis. 2016;10: 1–10. doi:10.1371/journal.pntd.0004844

39. Hashiguchi Y, Velez LN, Villegas N V., Mimori T, Gomez EAL, Kato H. Leishmaniases in Ecuador: Comprehensive review and current status. Acta Trop. 2017;166: 299–315. doi:10.1016/j.actatropica.2016.11.039

40. Medrano-Vizcaíno, P; Brito-Zapata, D; Rueda, A; Jarrín-V., P; García-Carrasco, JM; Medina, D; Aguilar, J; Acosta, N; Grilo, C; González-Suárez M. First national assessment of wildlife mortality in Ecuador: an effort from citizens and academia to collect roadkill data at country scale. Ecol Evol. 2023;In Press.

41. Medrano-Vizcaíno P, Grilo C, Silva Pinto FA, Carvalho WD, Melinski RD, Schultz ED, et al. Roadkill patterns in Latin American birds and mammals. Glob Ecol Biogeogr. 2022;31: 1756–1783. doi:10.1111/geb.13557

42. Grilo C, Koroleva E, Andrášik R, Bíl M, González-Suárez M. Roadkill risk and population vulnerability in European birds and mammals. Front Ecol Environ. 2020;18: 323–328. doi:10.1002/fee.2216

43. Sakata MK, Kawata MU, Kurabayashi A, Kurita T. Development and evaluation of PCR primers for environmental DNA (eDNA) metabarcoding of Amphibia. : 15–26. doi:10.3897/mbmg.6.76534

44. Breton AA, Beaty L, Bennett AM, Kyle CJ, Lesbarrères D, Torres S, et al.

Testing the effectiveness of environmental DNA (eDNA) to quantify larval amphibian abundance. 2022; 1229–1240. doi:10.1002/edn3.332

45. Goldberg CS, Strickler KM, Fremier AK. Science of the Total Environment Degradation and dispersion limit environmental DNA detection of rare amphibians in wetlands : Increasing ef fi cacy of sampling designs. Sci Total Environ. 2018;633: 695–703. doi:10.1016/j.scitotenv.2018.02.295

46. Haggerty CJE, Crisman TL, Rohr JR. Effects of forestry-driven changes to groundcover and soil moisture on amphibian desiccation, dispersal, and survival. Ecol Appl. 2019;29: e01870. doi:https://doi.org/10.1002/eap.1870

47. Hoffmann EP, Cavanough KL, Mitchell NJ. Low desiccation and thermal tolerance constrains a terrestrial amphibian to a rare and disappearing microclimate niche. Conserv Physiol. 2021;9. doi:10.1093/conphys/coab027

48. Hofstetter JR, Zhang A, Mayeda AR, Guscar T, Nurnberger JI, Lahiri DK. Genomic DNA from mice: A comparison of recovery methods and tissue sources. Biochem Mol Med. 1997;62: 197–202. doi:10.1006/bmme.1997.2637

49. Repolês BM, Gorospe CM, Tran P, Nilsson AK, Wanrooij PH. The integrity and assay performance of tissue mitochondrial DNA is considerably affected by choice of isolation method. Mitochondrion. 2021;61: 179–187. doi:10.1016/j.mito.2021.10.005

50. Wang MJ, Chen F, Lau JTY, Hu YP. Hepatocyte polyploidization and its association with pathophysiological processes. Cell Death Dis. 2017;8: e2805-7. doi:10.1038/CDDIS.2017.167

51. Koczur LM, Williford D, DeYoung RW, Ballard BM. Bringing back the dead:

Genetic data from avian carcasses. Wildl Soc Bull. 2017;41: 796–803. doi:10.1002/wsb.823

52. Flaherty BR, Barratt J, Lane M, Talundzic E, Bradbury RS. Sensitive universal detection of blood parasites by selective pathogen-DNA enrichment and deep amplicon sequencing. Microbiome. 2021;9: 1–19. doi:10.1186/s40168-020-00939-1

53. Sundar S, Singh OP. Molecular Diagnosis of Visceral Leishmaniasis. Mol Diagnosis Ther. 2018;22: 443–457. doi:10.1007/s40291-018-0343-y

Supporting information

S1 Fig. Gel electrophoresis of genomic DNA isolated from intestine samples. At the top of the electrophoresis is indicated the sample number. In the bottom is indicated the qualitative punctuation assigned for the analyses: “1” (minor degradation), “2” (medium degradation) o “3” (high degradation).

S1_raw_images. Raw data of gel electrophoresis. Contains the original photography of S1 Fig, Fig 5B and 6B.

Tables

S1 Table. Alternative regression models testing how DNA quantity and purity varied by class and time since death.

S2 Table. **Qualitative assignment of DNA degradation.** Samples were punctuated as

"1" (minor degradation), "2" (medium degradation) o "3" (high degradation).

Files

S1 File. **Database of samples analyzed in this study.** Samples were collected during a roadkill survey in the Napo region of Ecuador from 2020 to 2021.

Figure captions

Figure 1. Map of the study area in Ecuador. Study area in Napo province (Ecuador), marked by a red border square. The acronym SNAP (in Spanish) corresponds to Sistema Nacional de Áreas Protegidas. The black square marks Quito, the capital city of Ecuador.

Figure 2. DNA quantification by taxonomic class. Variation in the amount of DNA extracted from 817 tissue samples obtained from specimens of four vertebrate classes collected during roadkill surveys in the Napo region of Ecuador from 2020 to 2021. Different letters above the plot indicate significant differences between the groups.

Figure 3. Variation in the obtained quantity DNA from roadkill specimens at different estimated times since death across the four studied taxonomic classes. Symbols represent the observed data with the line and shaded area representing the estimated relationship and 95% CI respectively from a linear

regression model (Table 3). Samples were collected during a roadkill survey in the Napo region of Ecuador from 2020 to 2021.

Figure 4. DNA quantification by tissue. Variation in the amount of DNA extracted from different tissues collected from roadkill vertebrates during surveys in the Napo region of Ecuador from 2020 to 2021. Different letters above the plot indicate significant differences between the groups.

Figure 5. DNA integrity. Variation in the degradation of DNA extracted from intestine tissues collected from roadkill vertebrates during surveys in the Napo region of Ecuador completed in 2020-2021, correlated with the Time of Death (A) or the quantity (B). Different letters above the plot indicate significant differences between the groups.

Figure 6. Amplification of vertebrate molecular target. DNA electrophoresis from the indicated samples of vertebrates, containing isolated genomic DNA (A), or the amplified *COI* (B). Molecular weight (MW); Negative control (CN). The number of identification (ID) of each sample is shown in the top line.

Figure 7. Amplification of molecular target from *Leishmania* spp. DNA electrophoresis from the indicated samples of vertebrate's liver, containing isolated genomic DNA (A), or the amplified *18S SSU rRNA* of *Leishmania* spp.

(B).

Figure 1

Click here to access/download;Figure;Fig 1.tif

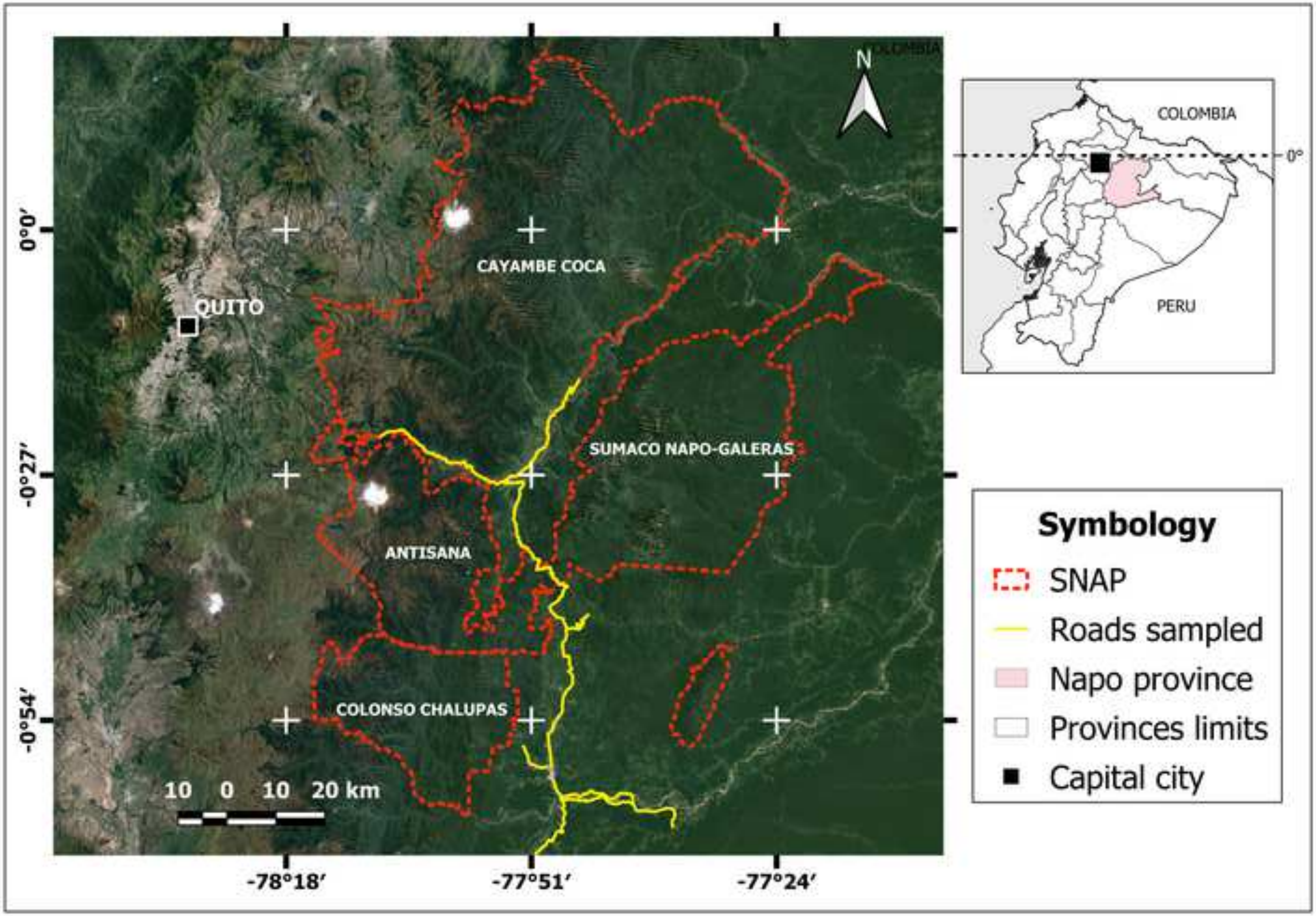

Figure 2

Click here to access/download;Figure;Fig 2.tif

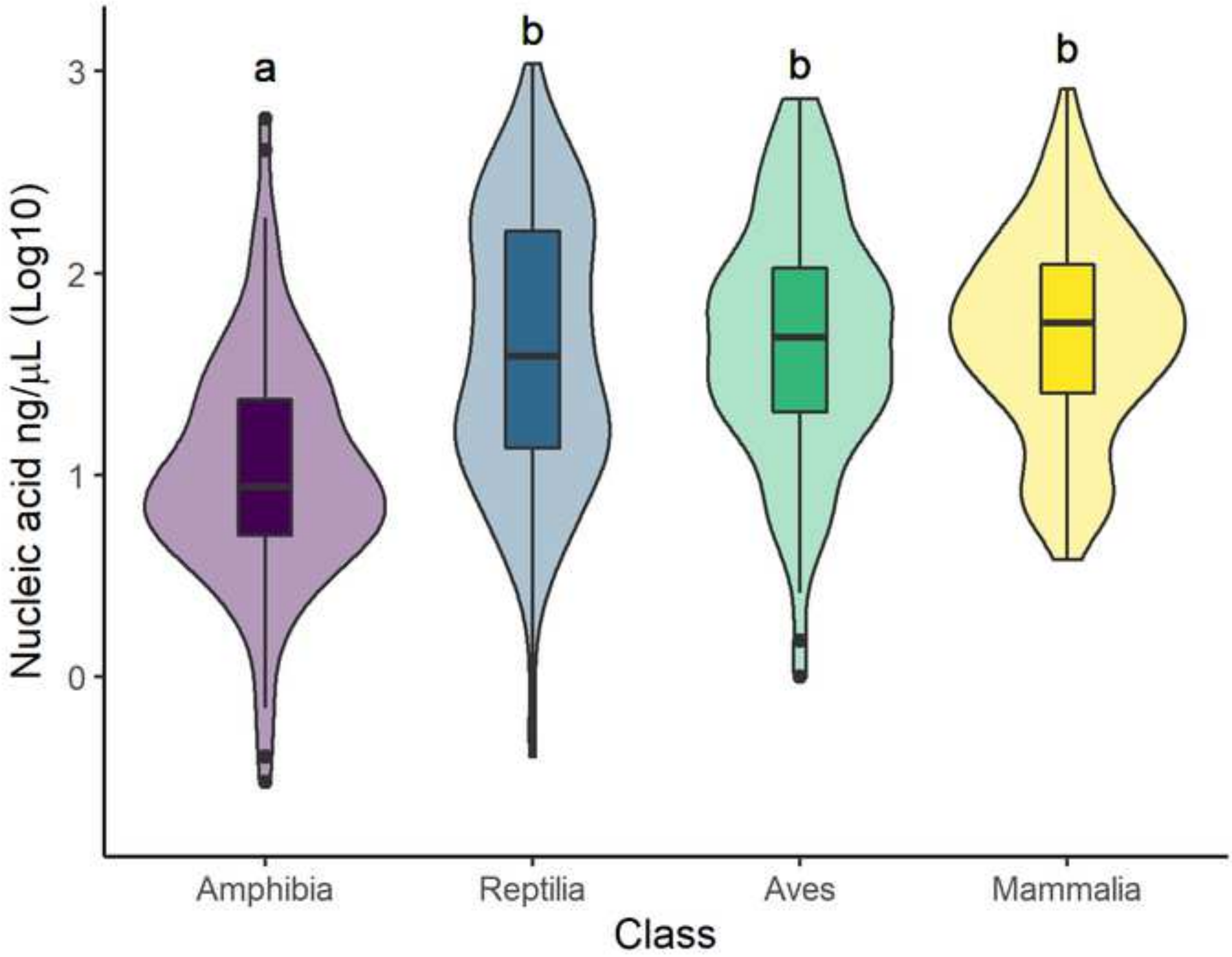

Figure 3

Click here to access/download;Figure;Fig 3.tif

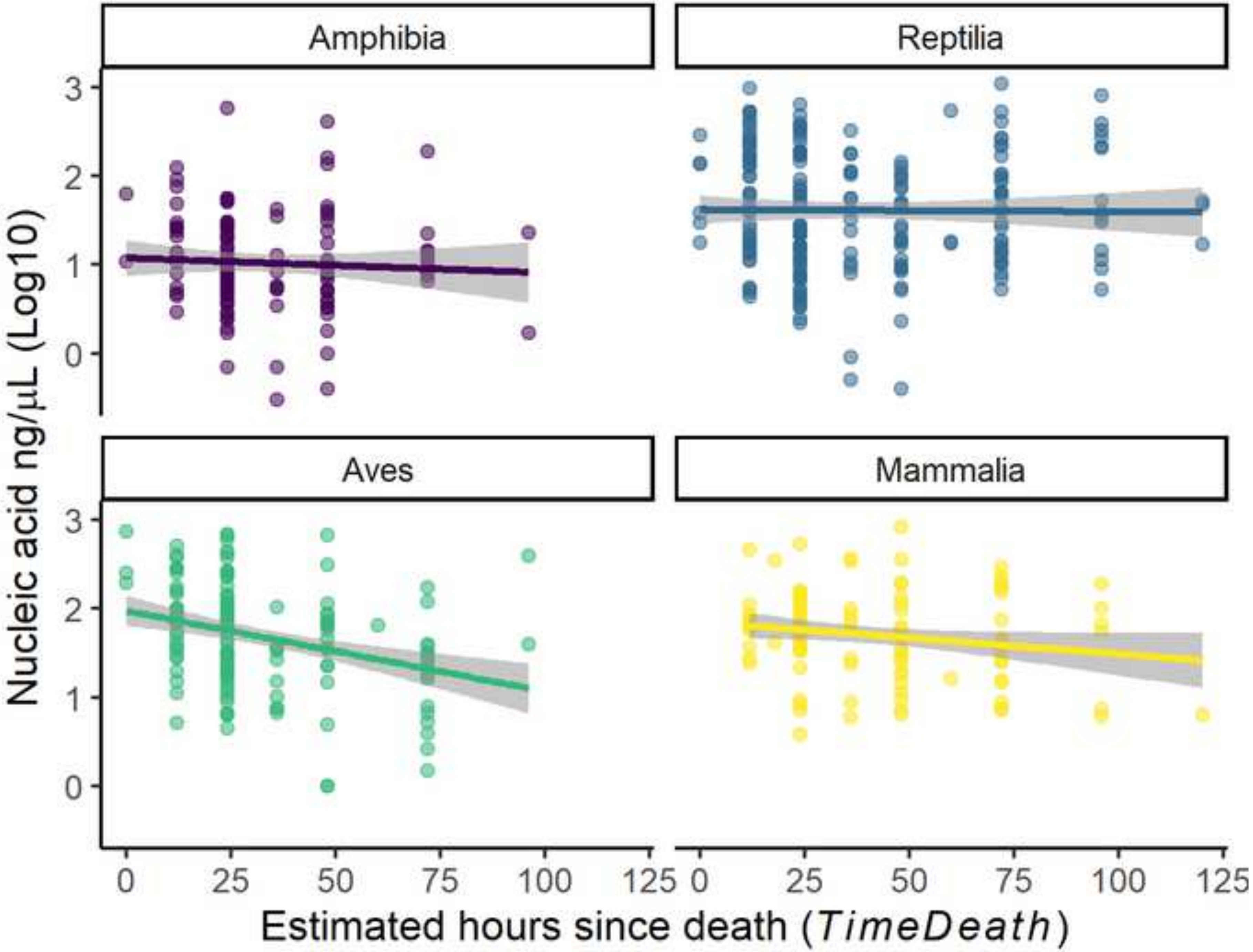

Figure 4

Click here to access/download;Figure;Fig 4.tif

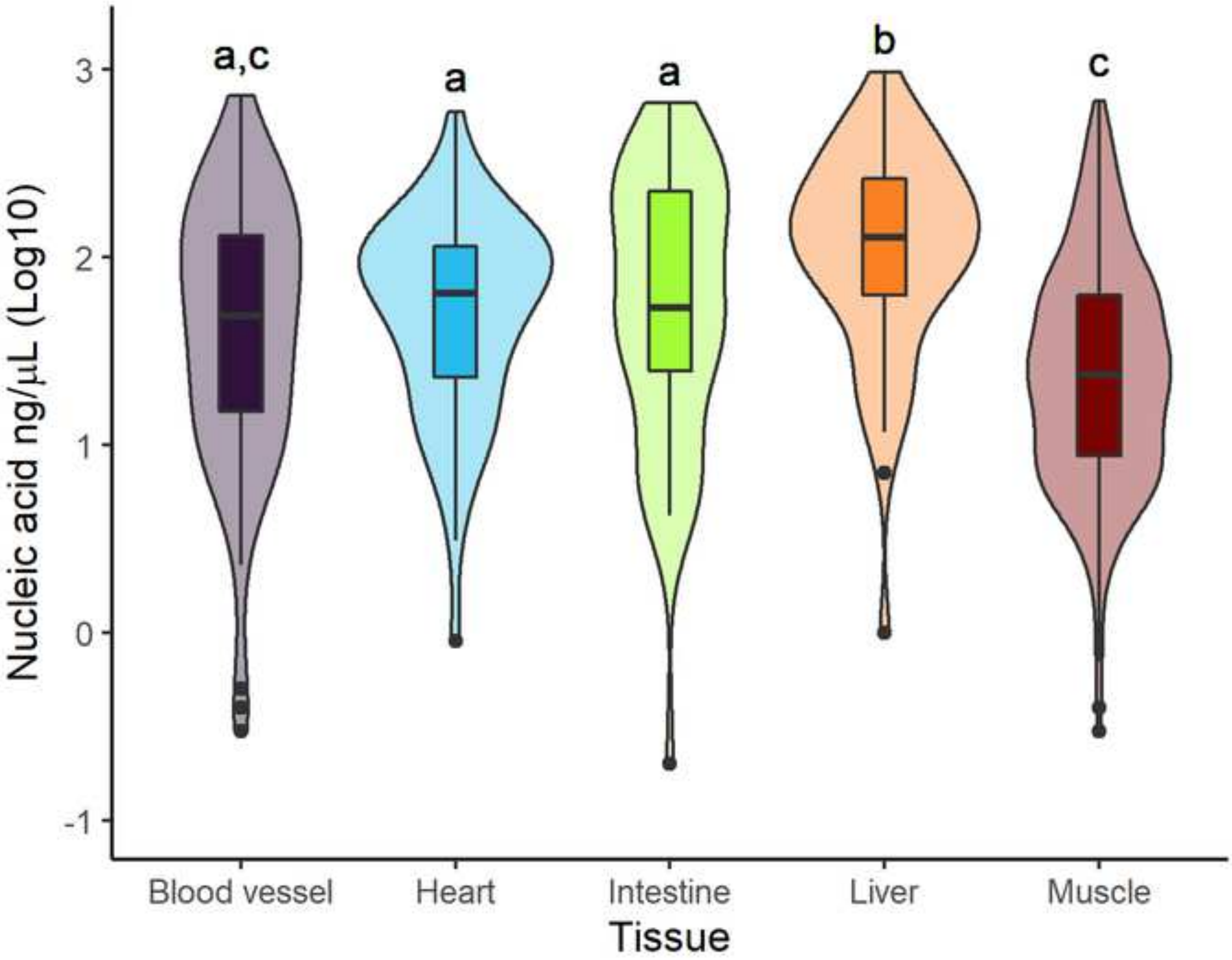

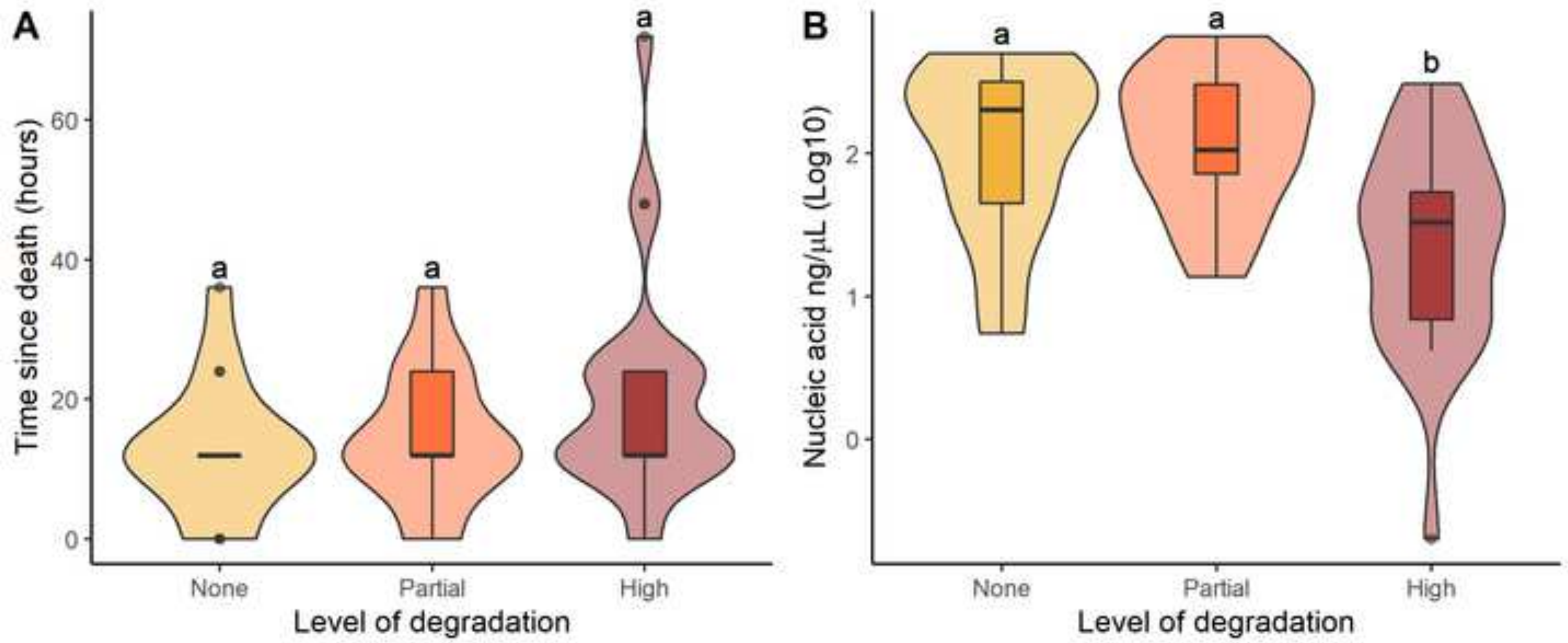
A
Time since death (hours)
0
20
40
60
a
a
a
None
Partial
High
Level of degradation
B
Nucleic acid ng/µL (Log10)
0
1
2
a
a
b
None
Partial
High
Level of degradation

Figure 6 Click here to access/download;Figure;Fig 6.tif

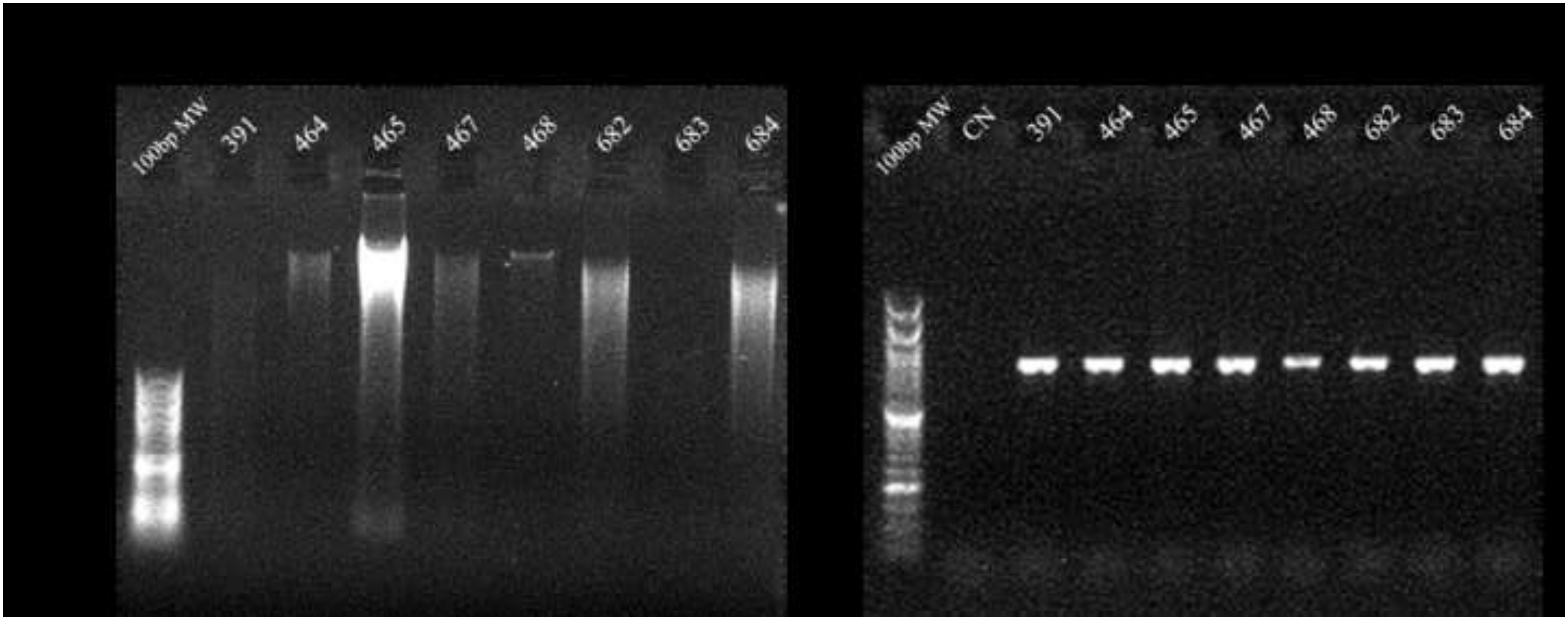

Figure 7

Click here to access/download;Figure;Fig 7.tif

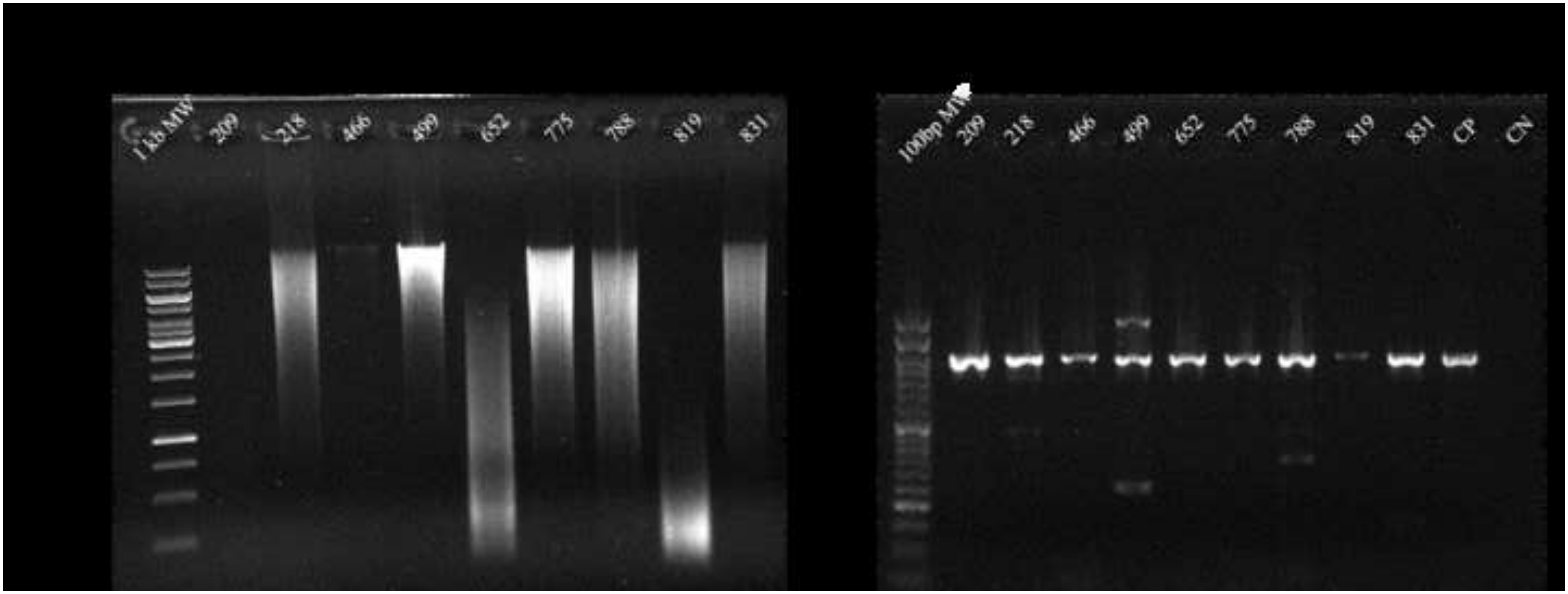

S1 Figure

Click here to access/download
Supporting Information
S1 Fig.tif

S1 File

Click here to access/download
Supporting Information
S1 File.xlsx

S1 Table

Click here to access/download
Supporting Information
S1 Table.docx

S2 Table

Click here to access/download
Supporting Information
S2 Table.docx

Click here to access/download
Supporting Information
S1_raw_images.pptx
