# Supplementary material for: From roads to biobanks: Roadkill animals as a valuable source of genetic data
Source: PLoS One. 2023 Dec 7;18(12):e0290836. doi: 10.1371/journal.pone.0290836 (PMC10703236; doi:10.1371/journal.pone.0290836)
Supplement: Attachment — Submitted filename: Response to the reviewers.docx [file pone.0290836.s008.docx]

PONE-D-23-09739R1

Dear Professor Kalendar,

Thank you for the review of our manuscript and the valuable comments provided for its improvements. We carefully reviewed the first round of reviews and then have spent two months dealing with an issue with copyright on the map, the last time we submitted it took a month to receive a response that during the multiple resubmission to address the map issue somehow this response letter was lost.

A point-by-point reply is provided below. We numbered the comments and start our replies with **Response:**

We hope this revised version is suitable for publication in PLoS ONE and look forward to hearing from you.

Manuela Gonzalez-Suarez on behalf of all authors.

**Revisions for the map (July/August 2023):**

Due to copyright issues flagged by PLoS ONE we are unable to include the map to show the study area, as this was not essential for the core message of the study, we have proceeded to remove the map and renumber figures in the manuscript.

**Editor comments and replies (May 2023):**

1. Please ensure that your manuscript meets PLOS ONE's style requirements, including those for file naming. The PLOS ONE style templates can be found at 
<https://journals.plos.org/plosone/s/file?id=wjVg/PLOSOne_formatting_sample_main_body.pdf> and 
<https://journals.plos.org/plosone/s/file?id=ba62/PLOSOne_formatting_sample_title_authors_affiliations.pdf>

**Response:** We check all style requirements. We hope not to have missed any details.

2. We suggest you thoroughly copyedit your manuscript for language usage, spelling, and grammar.

**Response:** Dr Manuela González-Suárez, who has studied and worked in universities in the USA and UK for over 15 years, has carefully revised the text for language use, spelling, and grammar.

3. Thank you for stating the following financial disclosure: 
"The authors would like to thank to: Corporación Ecuatoriana para el Desarrollo de la Investigación y Academia - CEDIA for the financial support given to the present research, development, and innovation work through its CEPRA program, especially for the Estudio de parásitos y microbioma de fauna silvestre en dos de las zonas más biodiversas del planeta: Los Andes Tropicales y Chocó-Darién en Ecuador fund (<https://cedia.edu.ec/servicio/fondo-idi-universidades/cepra-xvi-2022/>); Dirección de Investigación de la Universidad Central del Ecuador for Proyecto Senior 2021 fund (<https://www.uce.edu.ec/web/di>), and Reading University for SBS Seed Fund and a University International PhD studentship to PMV (<https://www.reading.ac.uk/>"

Please state what role the funders took in the study. If the funders had no role, please state: ""The funders had no role in study design, data collection and analysis, decision to publish, or preparation of the manuscript."" 
If this statement is not correct you must amend it as needed. 
Please include this amended Role of Funder statement in your cover letter; we will change the online submission form on your behalf.

**Response:** We agree to the financial disclosure:

"The authors would like to thank to: Corporación Ecuatoriana para el Desarrollo de la Investigación y Academia - CEDIA for the financial support given to the present research, development, and innovation work through its CEPRA program, especially for the Estudio de parásitos y microbioma de fauna silvestre en dos de las zonas más biodiversas del planeta: Los Andes Tropicales y Chocó-Darién en Ecuador fund (<https://cedia.edu.ec/servicio/fondo-idi-universidades/cepra-xvi-2022/>); Dirección de Investigación de la Universidad Central del Ecuador for Proyecto Senior 2021 fund (<https://www.uce.edu.ec/web/di>), and Reading University for SBS Seed Fund and a University International PhD studentship to PMV (<https://www.reading.ac.uk/>. The funders had no role in study design, data collection and analysis, decision to publish, or preparation of the manuscript."

4. PLOS ONE now requires that authors provide the original uncropped and unadjusted images underlying all blot or gel results reported in a submission’s figures or Supporting Information files.

**Response:** We have included the original images as file S1 raw images.

5. We note that you have stated that you will provide repository information for your data at acceptance. Should your manuscript be accepted for publication, we will hold it you provide the relevant accession numbers or DOIs necessary to access your data. If you wish to make changes to your Data Availability statement, please describe these changes in your cover letter and we will update your Data Availability statement to reflect the information you provide.

**Response:** We have now added File S2 (Sequences obtained in this study). All relevant data is available within the manuscript and its Supporting Information files.

6. Your ethics statement should only appear in the Methods section of your manuscript. If your ethics statement is written in any section besides the Methods, please move it to the Methods section and delete it from any other section. Please ensure that your ethics statement is included in your manuscript, as the ethics statement entered into the online submission form will not be published alongside your manuscript.

**Response:** Moved from Acknowledgments to the Methods section as required.

7. We note that Figure 1 in your submission contain map/satellite image which may be copyrighted.

**Response:** We have modified this figure to use a topography background available for which we provide the source citation: Jarvis A., H.I. Reuter, A. Nelson, E. Guevara, 2008, Hole-filled seamless SRTM data V4, International Centre for Tropical Agriculture (CIAT), <https://srtm.csi.cgiar.org>.

8. Please review your reference list to ensure that it is complete and correct. If you have cited papers that have been retracted, please include the rationale for doing so in the manuscript text, or remove these references and replace them with relevant current references. Any changes to the reference list should be mentioned in the rebuttal letter that accompanies your revised manuscript. If you need to cite a retracted article, indicate the article’s retracted status in the References list and also include a citation and full reference for the retraction notice.

**Response:** We have revised all references and all should be in order. As far as we area aware, we do not cite any retracted papers.

**Reviewer comments and replies:**

*Reviewer 1*

1) Generally looks good. So issues with the English (lack "," in many places) but this would not affect the quality of this study.

**Response:** Thanks. We have checked the whole document and corrected mistakes. We note that the use of commas is sometimes stylistic, like the Oxford comma. In those instances, we have followed our preference.

2) Line 68: add a "by" here maybe.

**Response**: added.

3) Line 97: by

**Response**: Corrected

4) Line 146: it might be better to know the version of R and the version of packages used in this study
5) Line 155: same with above:
6) Line 167: same with above:
7) Line 169: same with above:

**Response**: to all four comments above. Very good point, all versions now added in the text.

8) Line 298: just a minor suggestion: change the order for Table 7 and 8 because Table 8 was mentioned earlier than 7 om current text

**Response**: Done

9) Line 361: Suggest to delete this "However," it is not common to see two "However," in subsequent sentences.

**Response**: This section has been rewritten, removing both “However”

10) Line 367: if there is any data about the prevalence of such parasites among wildlife in that (or nearby) region that could be compared?

**Response**: To our knowledge, there is no data of *Leishmania* in wildlife or its prevalence in the same region of this study. In Ecuador there is very little information about natural Leishmania infections in wild fauna within the Pacific Coastal and Andes Regions. Only a few mammalian species have been reported as positive for Leishmania: *Sciurus vulgaris* *granatensis*, *Potos flavus*, *Tamandua tetradactyla*, *Choloepus hoffmani didactylus* and *Rattus rattus*, and also in some rodents (Reyes and Arrivillaga, 2009; Davies, et al., 2000; Hashiguchi et al., 2017; Hashiguchi et al., 2018).

Davies, C.R., Reithinger, R., Campbell-Lendrum, D., Feliciangeli, D., Borges, R., Rodriguez, N. 2000. The epidemiology and control of Leishmaniasis in Andean countries. Cad Saúde Pública. 16(4):925-50. doi: 10.1590/s0102-311x2000000400013.

Hashiguchi, Y., Velez, L.N., Villegas, N.V., Mimori, T., Gomez, E.A.L., Kato, H. 2017. Leishmaniases in Ecuador: Comprehensive review and current status. Acta Trop. 166:299-315. doi: 10.1016/j.actatropica.2016.11.039.

Hashiguchi, Y., Gomez, E.A.L., Cáceres, A.G., Velez, L.N., Villegas, N.V., Hashiguchi, K., Mimori, T., Uezato, H., Kato, H. 2018. Andean cutaneous leishmaniasis (Andean-CL, uta) in Peru and Ecuador: the vector Lutzomyia sand flies and reservoir mammals. Acta Trop. 178:264-275. doi: 10.1016/j.actatropica.2017.12.008.

Reyes, A., Arrivillaga, J. 2009. Fauna Mammalia asociada a los focos de leishmaniasis neotropical: Situación en Venezuela. Boletín de Malariología y Salud Ambiental, 49(1), 35-52. Recuperado en 11 de mayo de 2023, de http://ve.scielo.org/scielo.php?script=sci_arttext&pid=S1690-46482009000100002&lng=es&tlng=es

11) Line 373: delete this "an" maybe?

**Response**: Done

**Reviewer 2:**

1) The work is interesting, but the content examines the effects of using animals killed on the road for DNA isolation. The influence of the time since death and the type of tissue on the quality of the obtained DNA is being studied. However, there is no assessment of diversity, both between species and within species. So the work is very good, but it is necessary to change the title to be adequate to the content. There is no results or discussion about “monitor the diversity of vertebrates” and also only one microorganism was check in the DNA. May be something like "quality of DNA isolated from road killed animals depeding on time after death and kind of tissue" - or other because in this title there is nothing about microorganism.

**Response**: We agree and have changed the title to “From roads to biobanks: roadkill animals as a valuable source of genetic data”

2) The small comment about Line 112 – are values of precipitation and temperatures annual? –an average for a year? Or this values are for shorter period within year? The question is because of large differences.

**Response**: Thanks for this comment, values are annual means. Our study area is large and covered a wide altitudinal gradient, which also covers different conditions of precipitation and temperature. To make it clearer, we have modified this text in lines 114-117 to: “This area is located across an altitudinal gradient from 300 to 3000 m a.s.l., where the annual mean temperature varies from 4.63 to 23.7°C, and the annual precipitation from 1100 to 3400 mm. The study area is described in more detail in previous publications.
